# β-Sitosterol as a Promising Anticancer Agent for Chemoprevention and Chemotherapy: Mechanisms of Action and Future Prospects

**DOI:** 10.1016/j.advnut.2023.05.013

**Published:** 2023-05-27

**Authors:** Haoyu Wang, Zhi Wang, Zihui Zhang, Jingchun Liu, Li Hong

**Affiliations:** Department of Obstetrics and Gynecology, Renmin Hospital of Wuhan University, Wuhan, China

**Keywords:** β-sitosterol, phytosterol, phytochemical, cancer, chemoprevention, chemotherapy

## Abstract

Cancer is one of the primary causes of death worldwide, and its incidence continues to increase yearly. Despite significant advances in research, the search for effective and nontoxic preventive and therapeutic agents remains greatly important. Cancer is a multimodal disease, where various mechanisms play significant roles in its occurrence and progression. This highlights the need for multitargeted approaches that are not only safe and inexpensive but also provide effective alternatives for current therapeutic regimens. β-Sitosterol (SIT), the most abundant phytosterol found in various plant foods, represents such an option. Preclinical evidence over the past few decades has overwhelmingly shown that SIT exhibits multiple anticancer activities against varied cancers, such as liver, cervical, colon, stomach, breast, lung, pancreatic, and prostate cancers, in addition to leukemia, multiple myeloma, melanoma, and fibrosarcoma. In this article, we present the latest advances and perspectives on SIT—systematically summarizing its antitumor mechanisms of action into 7 main sections and combining current challenges and prospects—for its use as a promising agent for cancer prevention and treatment. In particular, SIT plays a role in cancer prevention and treatment mainly by enhancing apoptosis, inducing cell cycle arrest, bidirectionally regulating oxidative stress, improving metabolic reprogramming, inhibiting invasion and metastasis, modulating immunity and inflammation, and combating drug resistance. Although SIT holds such great promise, the poor aqueous solubility and bioavailability coupled with low targeting efficacy limit its therapeutic efficacy and clinical application. Further research on novel drug delivery systems may improve these deficiencies. Overall, through complex and pleiotropic mechanisms, SIT has good potential for tumor chemoprevention and chemotherapy. However, no clinical trials have yet proven this potential. This review provides theoretical basis and rationality for the further design and conduct of clinical trials to confirm the anticancer activity of SIT.

## Graphical abstract

**Figure:**
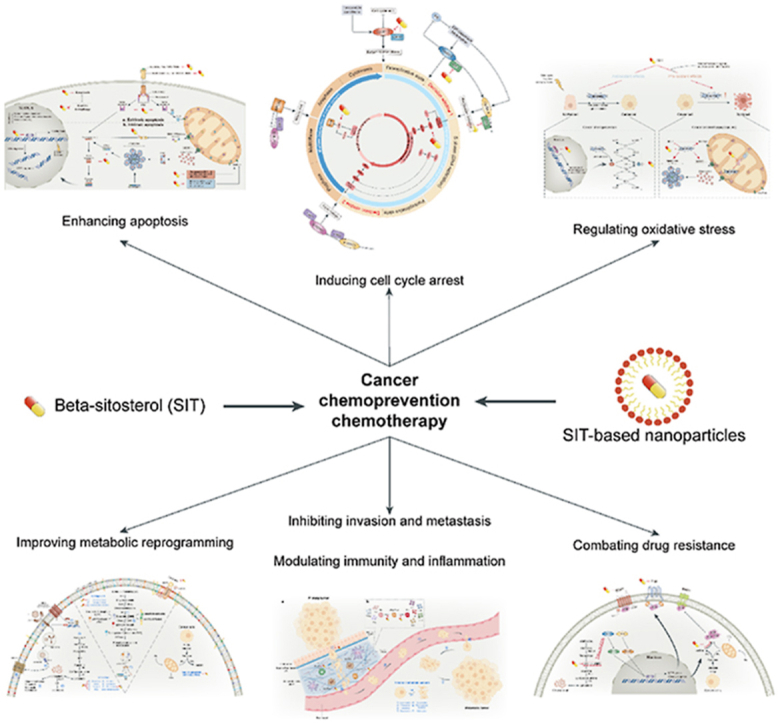

Seven main aspects of beta-sitosterol (SIT) antitumor mechanisms of action, and novel drug delivery systems for improving SIT deficiencies

**Statement of significance**

Innovatively, we systematically summarize the antitumor mechanisms of β-sitosterol (SIT) into 7 sections: enhancing apoptosis, inducing cell cycle arrest, bidirectionally regulating oxidative stress, improving metabolic reprogramming, inhibiting invasion and metastasis, modulating immunity and inflammation, and combating drug resistance. The major objective of this review was to provide a cohesive representation of the literature on the effects and mechanisms for dietary SIT cancer chemoprevention and chemotherapy and put forth theoretical basis, rationality, and suggestions for further research, particularly randomized controlled clinical trials, to validate the antitumor potential of SIT in humans.

## Introduction

Despite significant advances in research, cancer remains one of the primary causes of human deaths worldwide [1,2]. Based on the GLOBOCAN 2020 produced by the International Agency for Research on Cancer, the global cancer burden is expected to be 28.4 million cases in 2040, a 47% rise from 2020 [2]. The rapidly rising cancer burden urgently requires the development and implementation of novel, effective, and affordable chemoprevention and chemotherapy strategies to benefit diverse populations globally. In recent years, natural products such as phytochemicals have offered a broad platform for the development of new drugs against cancers. It is estimated that >60% of chemotherapeutic drugs are derived from phytochemical compounds, among which the most well-known are paclitaxel, docetaxel, etoposide, vincristine, and curcumin [3]. On the contrary, consumption of certain phytochemicals has been associated with reduced cancer risk, and the inherent safety and low cost make these “dietary drugs” an attractive option for widespread and long-term use in cancer chemoprevention [4]. Consequently, there has been growing interest in the identification and characterization of dietary phytochemicals with chemopreventive and chemotherapeutic properties and devoid of toxicity.

Phytosterols are essential steroids synthesized exclusively by plants. They resemble cholesterol in structure and function and comprise a major component of the human diet [5]. Several studies have reported that intake of phytosterol-rich diets can reduce risk of cancer to some extent [6]. β-Sitosterol (SIT) is the most abundant phytosterol and present in almost all plant foods and some traditional Chinese herbs. It has been applied in treating many diseases because of a broad range of biological functions, such as anti-inflammatory [7], antipyretic [8], analgesic [9] and antidiabetic [10] activities. Most importantly, it exhibits a significant anticancer potential [11]. In fact, SIT intake is partially responsible for the decreased incidence of prostate, colon, and esophagus cancers among vegetarians and men and women in Asian countries who consume much larger amounts of SIT than most Westerners [[12], [13], [14]]. In support of these epidemiologic studies, SIT feeding in rats helped prevent and reduce chemical carcinogen–induced abnormal crypts and colon tumors [15,16]. Controlled laboratory experiments at dietary relevant levels have also shown that SIT exhibits growth inhibitory and cytotoxic effects against a range of established cancer cell lines in vitro and in vivo but does not produce any acute/subacute toxicities [[17], [18], [19]]. Collectively, current preclinical evidence, such as in vitro studies, animal studies, and epidemiological investigations, supports SIT as a promising antitumor agent with chemoprevention and chemotherapy potential, although not yet confirmed through clinical trials.

As it is the case with most natural compounds, SIT has multitarget efficacy and systemic biological activities. It is widely known that the development of cancer is a multistep and multifactorial process and the prevention and treatment of cancer depends on multiple fronts and mechanisms [20]. Although the precise molecular mechanisms underlying the anticancer property of SIT has been elucidated to a certain extent, there has not been, to our knowledge, a systematic and up-to-date review focusing on its antitumor mechanisms. In this regard, based on a brief introduction on the structure, sources, and consumption of SIT, the current synopsis details the experimental evidence for the association of SIT with cancer prevention and treatment. Innovatively, we systematically summarize SITs antitumor mechanisms into 7 sections: enhancing apoptosis, inducing cell cycle arrest, bidirectionally regulating oxidative stress, improving metabolic reprogramming, inhibiting invasion and metastasis, modulating immunity and inflammation, and combating drug resistance. Some intrinsic connections between different sections are also proposed. Finally, we analyze the limitations that hinder the widespread use of SIT as an antitumor agent, such as poor bioavailability and solubility, and approaches to improve these limitations. The major objective of this review was to provide a cohesive representation of the literature on the effects and mechanisms for dietary SIT cancer chemoprevention and chemotherapy and put forth theoretical basis, rationality, and suggestions for further research, particularly randomized controlled clinical trials, to validate the antitumor potential of SIT in humans.

## Structure, Sources, and Consumption of SIT

**FIGURE 1:**
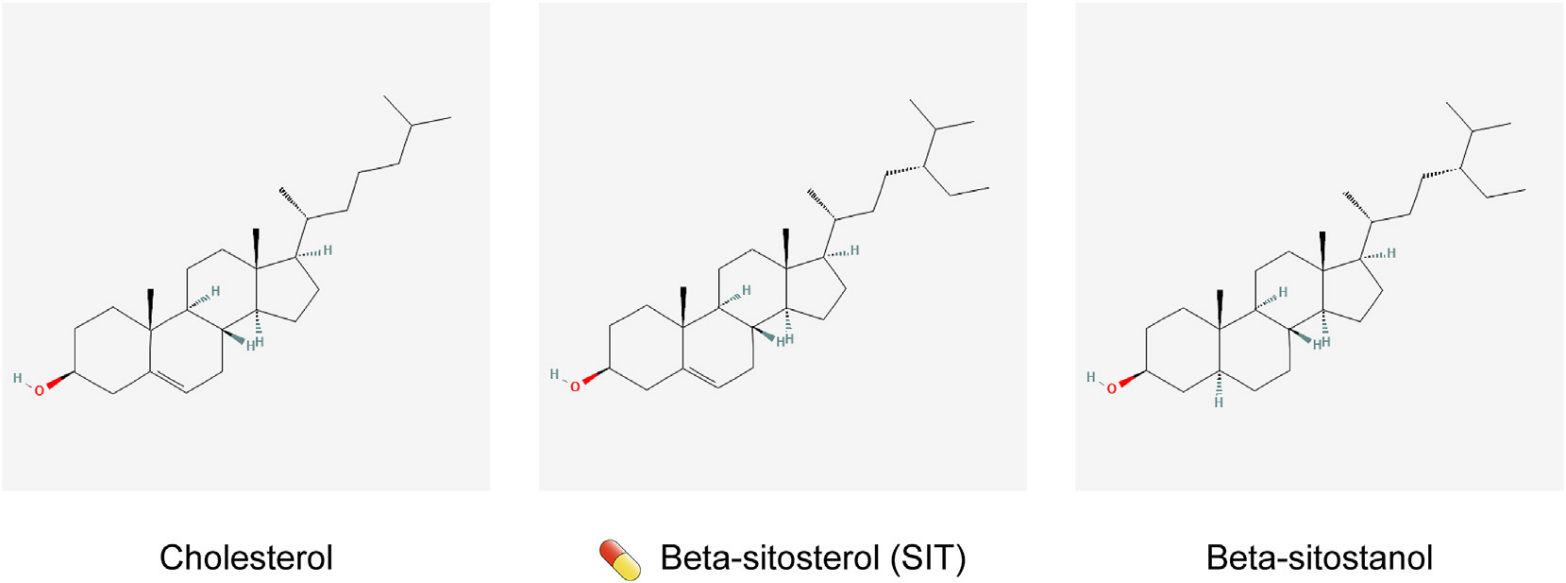
Chemical structure depictions of cholesterol, β-sitosterol (SIT), and β-sitostanol. The capsule-like symbol represents SIT.

Phytosterols are the counterparts of animal cholesterol. They are C-28 or C-29 sterols, differing from cholesterol (C-27) owing to presence of an extra hydrocarbon chain at the C-24 position (for SIT, it is an ethyl group) [5] (Figure 1). Phytosterols cannot be synthesized endogenously in the human body but are derived solely from the diet through intestinal absorption. Among dietary phytosterols, SIT accounts for ∼50%–65% [21]. SIT is enriched in plant foods such as nuts, peanuts, sesame seeds, soybean seeds, unrefined vegetable oils, and grains and in their products such as cornflakes, wheat bran, and wheat germ [22]. It is also present in some vegetables, fruits, and traditional medicinal herbs, such as pomegranate, avocado, *Cucurbita pepo*, saw palmetto, sea buckthorn, and wolfberries, although usually at lower concentrations when compared with nuts, legumes, and seeds [21]. Human dietary intake of phytosterols ranges from 40 to 400 mg/d, mainly depending on regional dietary habits and religious beliefs [23]. It is estimated that vegetarian diets and Asian diets represented by the Japanese diet contain 300–400 mg of phytosterols per day, whereas in Western diets, phytosterol intake is lower than 80 mg/d [23]. As opposed to SIT, Western populations consume higher concentrations of cholesterol than Asians. However, the oral bioavailability of SIT is lower than that of cholesterol. In humans, only 5%–10% of the total dietary SIT consumed is absorbed by the gut, whereas 45%–54% of the total cholesterol intake is absorbed [24]. Hence, SIT is found at concentrations 800–1000 times lower than that of endogenous cholesterol in the tissues and plasma of healthy people.

SIT is very stable, and drastic processes (such as boiling, bleaching, and deodorization) do not affect the SIT content in foods [21]. SIT can be chemically dehydrogenated to β-sitostanol, a fully saturated subgroup of phytosterols [5] (Figure 1). In addition to the free form, SIT exist in 4 forms of conjugates, in which the 3β-OH group is esterified to a fatty acid or a hydroxycinnamic acid or glycosylated with a hexose (usually glucose) or a 6-fatty-acyl hexose [5]. Because β-sitostanol and the conjugates occur in trace concentrations in many plant species and their antitumor activity has been poorly studied, they are not the subject of this review.

## Anticancer Mechanisms of SIT

Although not confirmed by clinical testing, a large number of in vitro and in vivo studies have shown that SIT has excellent anticancer potential. The exact mechanism by which SIT acts as a cancer prevention and treatment agent is still under investigation and is not thoroughly understood. However, a number of theories have been proposed, and the findings in these areas are summarized into the following 7 sections.

## Effects on apoptosis

**FIGURE 2:**
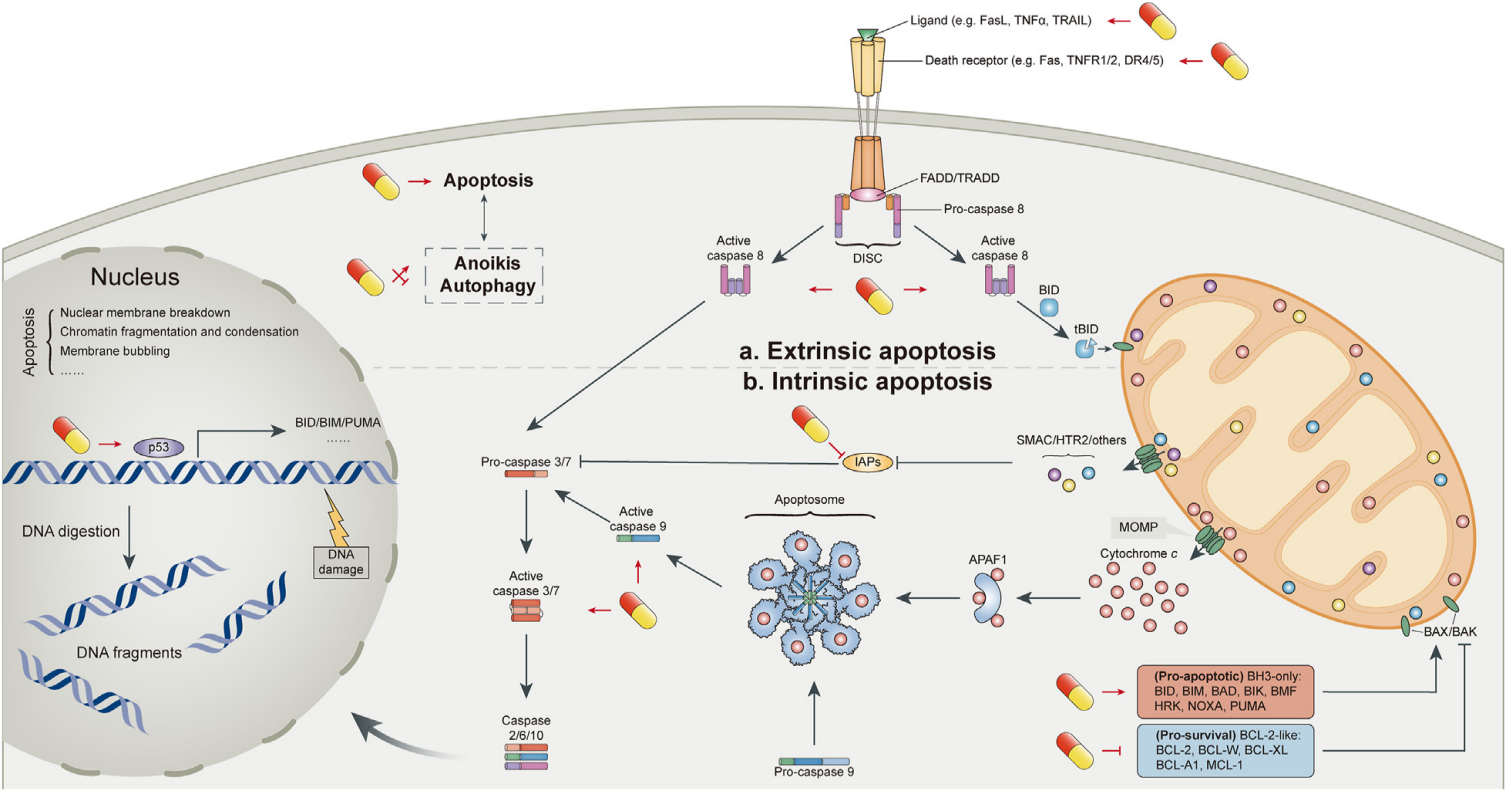
Apoptotic signaling pathways. Apoptosis can occur through 2 pathways: extrinsic (part **a**) and intrinsic (part **b**). The extrinsic (also called death receptor) apoptotic pathway involves the binding of a death receptor ligand to a member of the death receptor family. The intrinsic (also called mitochondrial) apoptotic pathway is induced by several different stimuli that unbalance the apoptotic rheostat and lead to mitochondrial outer membrane penetration (MOMP). The extrinsic and intrinsic pathways recruit and activate the initiator caspases 8 and 9, respectively, which cleaves and activates the executioner caspases 3 and 7 to complete wide-scale cleavage of cellular components and rapid cell death. Caspase 8–mediated cleavage and activation of BH3-only protein BID (to generate tBID) connects the extrinsic pathway to the intrinsic pathway. β-Sitosterol (SIT) promotes posttranslational activation of caspase 3/7/8/9, upregulates proapoptotic members and downregulates antiapoptotic members of the BCL-2 protein family by activating p53, blocks the inhibitory effect of IAPs on caspases, increases the expression of death receptors, and acts as a sensitizer for TRAIL-induced apoptosis. Additional cell death processes, such as autophagy and anoikis, are able to modulate apoptotic signaling pathways and are affected by SIT. The capsule-like symbol represents SIT. BCL, B cell lymphoma; IAP, inhibitor of apoptosis proteins; TRAIL, TNF –related apoptosis-inducing ligand.

For far too long, a vital target and vision of oncology has been the development of therapies that effectively promote the elimination of cancer cells through apoptosis because it is effective against many types of human cancer. However, deregulation of apoptosis is the hallmark of cancer, and agents that activate apoptosis in cancer cells could be valuable anticancer therapies [25]. As a major form of programmed cell death, apoptosis is mediated by 2 pathways: the intrinsic (mitochondrial) pathway and the extrinsic [death receptor (DR)] pathway. In addition, other signaling pathways interact with the apoptosis pathways to affect cell death. Through the enhancement of 2 apoptosis pathways and the regulation of related signaling pathways, SIT has become an excellent proapoptotic agent (Figure 2).

### Intrinsic apoptosis pathway

The intrinsic apoptosis pathway is finely regulated by the B cell lymphoma (BCL)-2 protein family, which consists of 3 subgroups: the antiapoptotic proteins, proapoptotic effectors, and proapoptotic BH3-only proteins. In response to stressors, such as DNA damage, growth factor withdrawal, and mitotic arrest, BH3-only proteins are activated by transcriptional upregulation and/or posttranslational modification. They subsequently activate BAX and BAK directly by binding or indirectly by neutralizing the antiapoptotic proteins (such as BCL-2 and BCL-X_L_) [26]. Activated BAX and BAK undergo oligomerization to form pores in the mitochondrial membrane, leading to mitochondrial outer membrane permeabilization (MOMP), the most critical event for triggering the mitochondrial apoptosis pathway [27]. In the context of MOMP, cytochrome c is released from mitochondria to bind apoptotic peptidase activating factor 1, forming a complex called apoptosome [28]. The apoptosome binds and cleaves caspase 9 preproprotein, releasing its mature, activated form. Activated caspase 9 stimulates the subsequent caspase cascade (caspase 3/6/7/10) that commits the cell to apoptosis [29]. Activation of this caspase cascade can be attenuated by inhibitor of apoptosis proteins (IAPs), such as XIAP, cIAP1, and cIAP2. MOMP also leads to the release of second mitochondrial activator of caspases and HTRA serine peptidase 2, both of which can functionally block IAPs, thus facilitating caspase activity [30]. The culmination of apoptosis is manifested by the breakdown of nuclear membrane and genomic DNA, membrane bubbling, and the cleavage of intracellular proteins (i.e., PARP and lamin) [27]).

A plethora of scientific studies are available pointing to the role of SIT in activating the intrinsic apoptosis pathway in a variety of cancers, such as liver cancer [[31], [32], [33], [34]], cervical cancer [35,36], colon cancer [[37], [38], [39], [40], [41]], stomach cancer [42,43], breast cancer [44,45], leukemia [46,47], multiple myeloma [48], lung cancer [49,50], melanoma [51], fibrosarcoma [52], pancreatic cancer [53], and prostate cancer [54] (Table 1). In these cultured cancer cell lines, SIT treatment causes posttranslational activation of caspases, release of cytochrome c, and proteolytic cleavage of PARP and genomic DNA. All these activities can be blocked by an inhibitor of caspase 3, confirming the promotion of apoptosis. Activation of the intrinsic apoptosis pathway by SIT is associated with an upregulation of BAX (or BAK) and downregulation of BCL-2 (or BCL-X_L_). The balance of antiapoptotic versus proapoptotic BCL-2 proteins has been considered as a cellular rheostat that controls the threshold of apoptosis [55]. In some cancers, SIT-induced upregulation of p53 contributes to the imbalance of apoptotic rheostat (Table 1). Several proapoptotic BCL-2 proteins, such as BAX, are transcriptional targets for p53 [56]. After exposure to various DNA-damaging agents, concentrations of these proteins will increase in cells expressing wild-type p53. This was particularly proved by the fact that SIT-induced apoptosis in A549 cells and NCI-H460 cells (p53 wild) but not in NCI-H23 cells (p53 mutant) and pifithrin-α (p53 inhibitor)–treated A549 cells [49]. Coincidentally, SIT is a potent DNA-damaging agent [42,49,50] shown by the comet assay, partly because it gives rise to excessive accumulation of reactive oxygen species (ROS), which will be discussed later. In addition, in some other cancers, the imbalance of apoptotic rheostat involves altered concentrations of IAPs (Table 1). During the process of carcinogenesis, IAPs and many antiapoptotic proteins, such as BCL-2, BCL-X_L_, and IAPs, are overexpressed in tumors to develop resistance against apoptotic death [25]. In this context, as a therapeutic agent of natural origin, SIT may play a similar role as the small-molecule inhibitors [57] or stapled peptides [58] currently synthesized to block these proteins.

**TABLE 1:** In vitro studies highlighting the activation of SIT on the intrinsic apoptosis pathway.

Cancer type	Cancer cell line(s)	Upregulated gene(s)	Downregulated gene(s)	Unaltered gene(s)	Signaling pathway(s)
Liver cancer [31]	HepG2, Huh7	Caspase 3/9	—	—	—
Liver cancer [32]	HepG2	Caspase 3	—	—	—
Liver cancer [33]	HepG2	BAX, caspase 3/9, p53	—	—	—
Liver cancer [34]	SMMC-7721	BAX, caspase 3/9	—	—	—
Cervical cancer [35]	Caski, HeLa	p53	—	—	—
Cervical cancer [36]	HeLa	Caspase 3/9	—	—	—
Colon cancer [37]	HT-115	BAX, caspase 3, p53, p21	BCL-2, PCNA, Mdm2	—	—
Colon cancer [38]	COLO 320, DM		β-catenin, PCNA	—	—
Colon cancer [39]	HT-116	BAX, caspase 3/9	BCL-2	—	—
Colorectal cancer [40]	HCT116	BAX, BAK, caspase 3, p53	BCL-2	—	—
Colon cancer [41]	HT-29	Caspase 3	—	—	—
Stomach cancer [42]	SGC-7901	BAX, caspase 3	BCL-2, cIAP-1	cIAP-2	—
Stomach cancer [43]	SNU216, SNU601, AGS	Caspase 3/7	—	—	AMPK/PTEN/HSP90
Breast cancer [44]	MDA-MB-231	BAX	BCL-2	—	ERK1/2
Breast cancer [45]	MDA-MB-231	Caspase 3/8/9	—	—	—
Leukemia [46]	U937	Caspase 3	BCL-2	BAX, BCL-X_L_, cIAP-1, cIAP-2	—
Leukemia [47]	HL-60	Caspases 3/9	—	—	—
Multiple myeloma [48]	U266	Caspase 3	BCL-2, BCL-X_L_	—	AMPK/ACC; Akt/mTOR; JNK
Lung cancer [49]	A549, NCI-H460	BAX, caspase 3/9, p53, p21	BCL-2	—	—
Lung cancer [50]	A549	BAX, caspase 3/9	BCL-2	—	—
Melanoma [51]	H1_DL2	Caspase 3	—	—	—
Fibrosarcoma [52]	MCA-102	BAX, p53, p21	BCL-2, XIAP, cIAP-1, cIAP-2	—	Akt/mTOR; p38 MAPK
Pancreatic cancer [53]	MIAPaCa-2, BXPC-3	BAX	BCL-2	—	NF-κB
Prostate cancer [54]	PC-3, DU-145	BAX	BCL-2	—	—

### Extrinsic apoptosis pathway

The extracellular ligand–induced DRs signaling are central to the extrinsic apoptosis pathway. DR is a class of cell membrane proteins, whose well-characterized members include Fas [59], TNF receptors TNFR1 and TNFR2 [60], and TNF–related apoptosis-inducing ligand (TRAIL) receptors DR4 and DR5 [61]. On binding and activation by its cognate ligand (FasL, TNF-α, or TRAIL, respectively), the DR oligomerizes to form flatforms at the cell surface. This process leads to the recruitment of downstream adaptor proteins, such as FAS-associated death domain (FADD) or TNFR1-associated death domain (TRADD), and initiator caspases, such as caspases 8 and 10, to form the death-inducing signaling complex [62]. The assembled death-inducing signaling complex subsequently activates effector caspases (i.e., caspases 3, 6, and 7) to unleash the downstream demolition process of apoptosis [63]. In the extrinsic pathway, the activation of caspase 8 is negatively regulated by FADD-like apoptosis regulator [64]. In addition, activated caspase 8 can convert BH3-only protein BID into the proapoptotic form, tBID, which acts on the proapoptotic BAX-BAK molecular switch, leading to MOMP [65]. Thus, BID has been proposed as an intimate connection between the intrinsic and extrinsic pathways.

The modulation of SIT on the extrinsic apoptosis pathway has been proved in several cancer types. After the treatment with Sanyeqing petroleum ether component, with SIT as the main ingredient, the activity of caspase 8 in HeLa cells was significantly strengthened, indicating the activation of the extrinsic pathway [36]. Similarly, SIT stimulation could increase Fas expression and caspase 8 activity in breast cancer cells, which was responsible for the inhibition of cell growth [66]. Unlike TNF receptors, activation of TRAIL receptors generally causes no severe systemic inflammatory responses, providing a convincing rationale to induce cancer apoptosis with TRAIL receptor agonists [67]. However, recent studies have shown that many cancers are resistant to TRAIL agonists or recombinant TRAIL-activating monoclonal antibodies, leading researchers to focus on combination therapies such as sensitizers for TRAIL-induced apoptosis. Park et al. [68] noted that SIT is an effective sensitizer in TRAIL-resistant breast cancer cells. Compared with treatment using SIT or TRAIL alone, synergistic treatment with subtoxic concentration of the 2 significantly attenuated cell viability increased caspase 3/8/9 activity and remarkably upregulated the proapoptotic BAX and downregulated the antiapoptotic XIAP. It is suggested that SIT may serve as a promising TRAIL sensitizer to enhance TRAIL-mediated cancer cell death.

### Apoptosis-related signaling pathways

In addition to the antiapoptotic members of the BCL-2 family, IAP proteins, and FADD-like apoptosis regulator, many signaling pathways influence the death phenotype of tumor cells. Examples include kinase signaling pathways involving AMPK [69], PI3K/AKT/mTOR [70], RAS/RAF/MAPK [71], and JAK/STAT [72]. Tumor cells become “addicted” to these pathways because they interact with the mediators of apoptosis pathways, thereby inactivating cell death. For example, activation of AKT signaling induces BAD phosphorylation and inhibits apoptosis [73]. Hence, a number of signaling pathways promoting cell survival have become established therapeutic targets in oncology. Current studies suggest that the proapoptotic effect of SIT involve the regulation of ≥3 signaling pathways. First, cancer metabolism-related AMPK signaling pathway plays an important role in SIT-induced apoptosis. Moreover, SIT upregulated AMPKα in breast cancer cells [74]. In multiple myeloma, SIT increased the phosphorylation of AMPK and its substrate ACC in a dose-dependent manner, and the AMPK inhibitor compound C notably prevented caspase 3 activation induced by SIT [48]. Furthermore, SIT exerted anticancer effects on gastric adenocarcinoma cells in vitro and in vivo through AMPK/PTEN and AMPK/HSP90 axes [43]. Second, the proapoptotic effect of SIT is mediated partly through the block of PI3K/AKT/mTOR signaling pathway. This is demonstrated by the fact that SIT attenuated the phosphorylation of AKT/mTOR in different cancer cell lines, and PI3K inhibitor LY29004 significantly augmented SIT-induced cell death [48,52,74]. In addition, SIT inhibited the NF-κB pathway [53]. As a downstream effect of AKT pathway activation, the NF-κB pathway enables tumor cells to escape apoptosis through the upregulation of prosurvival genes. Third, the MAPK signaling pathways, consisting of 4 subfamilies (ERK, JNK, p38 MAPK, and ERK5), are affected by SIT. Vundru et al. [44] and Moon et al. [52] demonstrated that SIT preferentially activated ERK1/2 and p38 MAPK, but not JNK, for its cell death–inducing effect in cancer cells. However, Sook et al. [48] reported the opposite result that SIT-induced apoptosis in multiple myeloma was solely mediated by JNK, excluding regulatory effects on ERK or p38 MAPK. In summary, SIT promotes apoptotic death of cancer cells by regulating multiple upstream signaling pathways, which undoubtedly makes it promising in the treatment of cancer.

### Other forms of programmed cell death

Although most studies have focused on apoptosis, 2 separate studies have reported the role of SIT in other forms of programmed cell death, such as autophagy and anoikis. Autophagy is a regulated process that responds to signals from the tumor microenvironment (TME). Recent studies revealed the paradoxical nature of autophagy in determining cell-fate machinery: autophagy induces cell death, inhibits inflammation, and enhances genomic stability; conversely, autophagy also enables cells to survive under stressful conditions and is believed to be a prosurvival mechanism [75]. In non–small-cell lung cancer (NSCLC), SIT treatment suppressed autophagy flux and viability of A549 cells in vitro and in vivo through inactivating the TGF-β/Smad2/3/c-Myc pathway [76]. These results indicate that the inhibitory effect of SIT on autophagy may serve as a novel target for the treatment of NSCLC. Apoptosis caused by the loss of normal cell–matrix interactions is called anoikis. It represents a particular mode of apoptosis, namely a wide range of cellular responses to loss of adhesion that use diverse signaling and apoptotic pathways [77]. SIT, which has been shown to have anticancer effects in colorectal cancer, induced anoikis by inhibiting the EGFR/AKT signaling pathway [40]. This provides a basis for the potential use of SIT as an alternative anticancer agent for anoikis resistance–related invasion and metastasis of colorectal cancer.

### Effects on cell cycle

**FIGURE 3:**
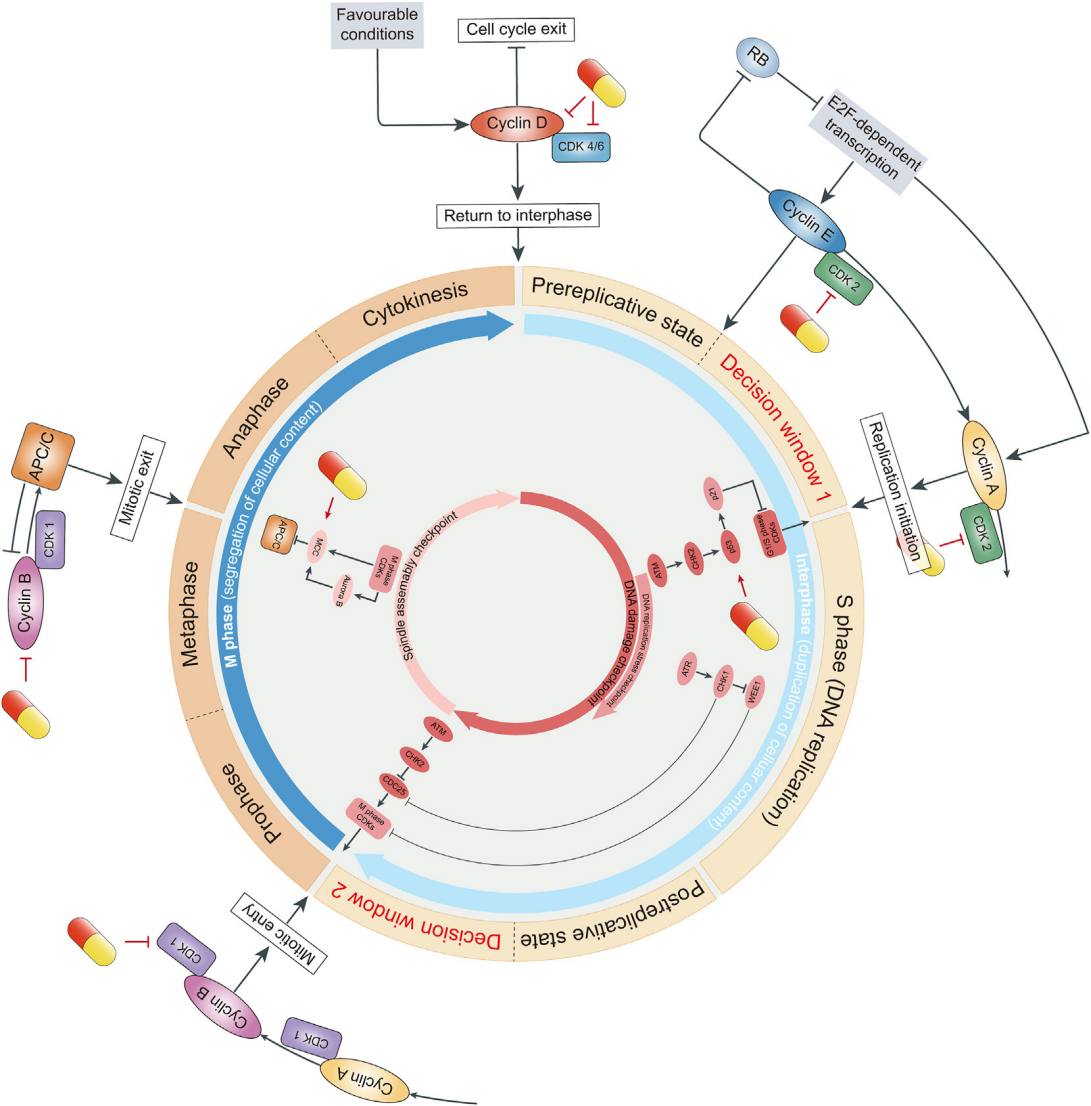
Cell division cycle and cell cycle control. Rings: The blue ring shows the cell division cycle, such as interphase (light blue) and M phase (dark blue). The peripheral orange-yellow ring shows the specific division of interphase and M phase and 2 decision windows. Outside the rings: Cyclin CDKs drives cell cycle progression. Under favorable conditions, the accumulation of cyclin D-CDK4/6 activity allows entry into the cell cycle, thereby preventing cell cycle exit. E2F-dependent transcription leads to the accumulation of cyclins E and A, which creates a decision window to enter S phase. Cyclin E-CDK2 activity further activates E2F-dependent transcription, forming a positive feedback loop that leads to increased activities of cyclin E-CDK2 and cyclin A-CDK2. This process allows the accumulation of cyclin A-CDK2 activity and S entry through the inactivation of APC/C^CDH1^ activity (not shown). Subsequent accumulation of cyclin A/B-CDK1 creates the second decision window for mitotic entry. Accumulation of cyclin A/B-CDK1 activity drives mitotic entry and allows APC/C^CDC20^ activation, which is required for mitotic exit and targeted degradation of cyclins to complete the cell cycle. β-Sitosterol (SIT) has an inhibitory effect on the activity of various cyclin–CDK complexes, such as cyclin D and CDK2, 4, and 6. Inside the rings: In response to DNA damage during interphase, replication stress during S phase, and abnormal spindle assembly during M phase, specific cell cycle checkpoints block or slow down the cell cycle by inhibiting CDKs activity and the APC/C. The capsule-like symbol represents SIT. APC/C, anaphase-promoting complex/cyclosome.

The cell cycle dysregulation and continual cell division is a hallmark of cancer, commonly resulting from mutations or malfunction of cell cycle control pathways, such as cyclins, CDKs, and checkpoint control proteins. One mechanism by which SIT function as a cancer chemopreventive and chemotherapeutic agent is interfering with the continuous cell cycle progression of tumor cells (Figure 3).

### Modulation on cyclin CDKs

The mitotic cell cycle is divided into 4 distinct phases, namely, G1, S, G2, and M phases. The S phase is the stage where DNA replication occurs, whereas the M phase serves the dual function of precisely separating the duplicated DNA and splitting the entire cellular content into 2 daughter cells. Prereplicative G1 phase and postreplicative G2 phase are 2 intervals separating the S phase from the M phase. They are critical periods for cell cycle regulation because of 2 decision windows: one in G1 during which cells can commit to initiate DNA replication and enter the cell cycle, and the other in G2 during which cells can commit to initiate chromatin aggregation and the central alignment of chromosomes to complete the cell division.

The key regulator of cell cycle is CDKs, whose activity depends on the regulatory subunit, cyclins. Cyclin–CDK activities are closely related to the normal progression of cell cycle. Early in the G1 phase, cells face a “choice” to either remain in a cell cycle state or exit the cell cycle into quiescence. Cyclin D-CDK4/6 plays a central role in this decision [78]. In response to various mitotic stimuli, cyclin D-CDK4/6 creates a transition state between cell cycle exit and the first decision window by controlling the metabolic state of the cell [79]. Inhibition or absence of CDK4/6 activity can cause cells to exit the G1 phase into quiescence. During the transition state, hyperphosphorylation and inactivation of retinoblastoma protein by cyclin E-CDK2 activates 17β-estradiol (E_2_)F-dependent transcription, initiating a positive feedback loop that promotes cells into the first decision window [78]. Once the decision has been made to enter a new cell cycle, activation of E_2_F-dependent transcription and inactivation of APC/C^CDH1^ allow cells to accumulate sufficient cyclin A-CDK2 activity to initiate DNA replication [80]. In another decision window for the commitment to enter mitosis, CDK1 plays a central role. Cyclin A/B that accumulates gradually from the S phase binds to and activates CDK1, and once CDK1 activity reaches the threshold level, the entry into mitosis will be triggered by widespread phosphorylation of thousands of CDK1 substrates [81]. Mitotic phosphorylation induces structural changes in each cell compartment and primes the cell for DNA separation and division. Activated cyclin B-CDK1 also leads to the activation of APC/C^CDC20^, which promotes proteolytic destruction of cyclin B and initiates mitotic exit at metaphase. In addition, the activation of APC/C^CDC20^ triggers sister chromatids separation and cell division, ultimately resetting the cell cycle in both daughter cells to the pre-G1 phase [82].

Because the primary driver of cell cycle progression is CDKs activity and increased cyclin CDKs has been widely reported in cancers, CDKs have become attractive targets for new cancer therapies [83]. CDK inhibitors can prevent continual cell cycle progression by forcing cancer cells to permanently exit the cell cycle into senescence or apoptosis. As a natural compound with multiple targets, SIT modulates the activity of diverse cyclin–CDK to induce cell cycle arrest (Supplemental Table 1). In NSCLCs [49], SIT downregulated the expression of cyclin D-CDK4 and cyclin D-CDK2, whereas in cancers of the oral cavity and pharynx [84], SIT inhibited the expression of cyclin B. Accordingly, SIT blocked the cell cycle progression of these cancer cells at the G0/G1 and G2/M phases, respectively. In breast cancer, SIT can either downregulate cyclin D-CDK4, inducing G0/G1 arrest, or inhibit CDK1, inducing G2/M arrest [44,85]. Because there is a specific role for cholesterol in regulating the activity of CDK1 [86], the cholesterol-starving effect of SIT (discussed later) may contribute to the cell cycle arrest. In addition, SIT upregulated p27, which uses several effects on the cell cycle including inhibition of cyclin D-CDK4/6 [44]. However, in other cancer types where SIT induce cell cycle arrest, whether and what type of cyclin–CDK activity is affected remains to be further investigated [41,50,53,76,87].

### Modulation on cell cycle checkpoints

Cells rely on cell cycle checkpoints to prevent the accumulation and spread of genetic errors during cell division. These cell cycle control checkpoints include the DNA damage checkpoint, the DNA replication stress checkpoint, and the spindle assembly checkpoint (SAC). Because the cell cycle is a finely regulated process, endless cycles of division present a fundamental challenge to cancer cells that require these checkpoints to remain functional.

Throughout the cell cycle, the occurrence of DNA damage triggers rapid signaling responses that depend on the DNA damage checkpoint protein kinase ataxia telangiectasia mutated (ATM). These responses lead to mobilization of DNA repair machinery and interaction with cell cycle regulators, resulting in slowing or arresting of progression through the cell cycle to prevent the accumulation and spread of genetic errors during cell division [88]. Downstream of ATM, key targets for cell cycle control includes the protein kinase CHK2 and the transcription factor p53. In the G1 phase, p53 activates the CDK inhibitor p21, which leads to the inhibition of cyclin–CDK complexes primarily in the first decision window, thereby preventing S phase entry [89]. In the S and G2 phases, CHK2 degrades CDC25, thus reinforcing WEE1-dependent inhibitory phosphorylation of CDK1 to prevent mitotic entry [90]. Unlike the DNA damage checkpoint, the DNA replication stress checkpoint protein kinase ataxia telangiectasia and Rad3-related (ATR) and its downstream effector CHK1 function only during the S phase. Their main function is delaying mitotic entry to allow more time for replication to be completed, thereby preventing replication stress–induced DNA damage. Mechanically, ATR restricts CDK activities mainly through CHK1-dependent CDC25 degradation and WEE1 activation to control cell cycle progression [88]. DNA replication stress response and DNA damage response are intertwined. Replication stress–induced DNA damage eventually triggers the activation of DNA damage checkpoint that inhibits early tumor development by preventing proliferation.

As described, the DNA damage checkpoint is critical to initiate quiescence, senescence, or programmed cell death primarily through the p53/p21 axis-dependent pathway in the face of irreparable DNA damage. However, p53 mutations are the most common mutations in cancers, which impair their ability to exit the cell cycle and promote genomic instability and tumor progression [91]. Therefore, the p53/p21 axis is getting more attention in anticancer chemotherapy. Studies have shown that SIT has a promoting effect on the p53/p21 axis. The inhibition of CDKs activities and induction of cell cycle arrest by SIT partly comes from the upregulation of p53 and p21 (Supplementary Table 1), which ultimately results in apoptosis of cancer cells. Although no studies have explored whether SIT simultaneously modulates other molecules involved in the DNA damage response, such as ATM, CHK2, CDC25, or WEE1, these results at least illustrate a potential stabilizing and protective effect of SIT on the DNA damage checkpoint. On the contrary, owing to the presence of persistent oncogene-induced replication stress, cancer cells rely heavily on the functional replication stress checkpoint response to ensure normal completion of DNA replication [92]. Future research could focus on the ability of SIT to target the DNA replication stress tolerance in cancer and try to combine SIT with ATR and CHK1 inhibitors for chemotherapy.

SAC performs surveillance functions during the M phase to ensure correct chromosome segregation and an equal distribution of replicated DNA between the 2 daughter cells [93]. The SAC machinery relies on the mitotic checkpoint complex (MCC) (consisting of MAD2, BUBR1, and CDC20), which is recruited to any kinetochores not bound to microtubules after phosphorylation by Aurora B and CDK1 [93]. Only when all kinetochores are bi-attached does the lack of SAC activity leads to disassembly of MCC and release of CDC20, which acts as a coactivator of APC/C to initiate anaphase. Otherwise, SAC acts as a “delay” signal that maintains APC/C inhibition and M phase arrest [94]. If chromosome biorientation is not resolved after prolonged mitotic arrest, cells will follow 2 paths: apoptosis through caspase activation or exit from the M phase as a single tetraploid cell [95]. In cancer, compromised cell cycle exit checkpoints set the stage for continuous cell division, potentially leading to a greater reliance on SAC. Complete removal of SAC in cancer cells results in catastrophic chromosome loss that is absolutely lethal [96].

Microtubule cytoskeleton, whose major component is tubulin, is required for normal biorientation of kinetochores and segregation of chromosome [97]. Two well-known microtubule-targeted chemotherapeutic drugs, vinca alkaloids and taxanes, exert antitumor effects by inhibiting and promoting microtubule polymerization, respectively, to disrupt mitotic spindle formation [98]. The failure of mitotic spindle formation keeps SAC active and, thus, induces cell cycle arrest and apoptosis. Recently, some studies have demonstrated that SIT can also act as a microtubule-targeted agent. In leukemia, SIT promoted microtubule polymerization through an increase of polymeric α-tubulin, thereby inducing G2/M arrest [99]. A large population of arrested cells subsequently underwent endoreduplication and eventually progressed to apoptosis. This disruption of microtubule dynamics by SIT was closely related to BCL-2 phosphorylation and PI3K/AKT signaling pathway activation. In another study, by binding to a novel site, SIT can interact directly with tubulin to stabilize microtubule assembly in a Taxol-like manner [100]. Combination of the 2 microtubule-stabilized drugs, SIT and Taxol, has potential to increase the anticancer efficacy of individual drugs, whereas minimizing toxic side effects. In addition, SIT inhibited microtubule polymerization in cervical cancer by downregulating α-tubulin and microtubule-associated protein 2 [101]. The abovementioned studies suggest that SIT can either stabilize or destabilize microtubules to prevent cancer cells from forming bipolar spindles, consequently killing cancer cells in virtue of the SAC machinery. It may make sense to further investigate whether SIT has direct modulations on the MCC.

### Effects on oxidative stress

**FIGURE 4:**
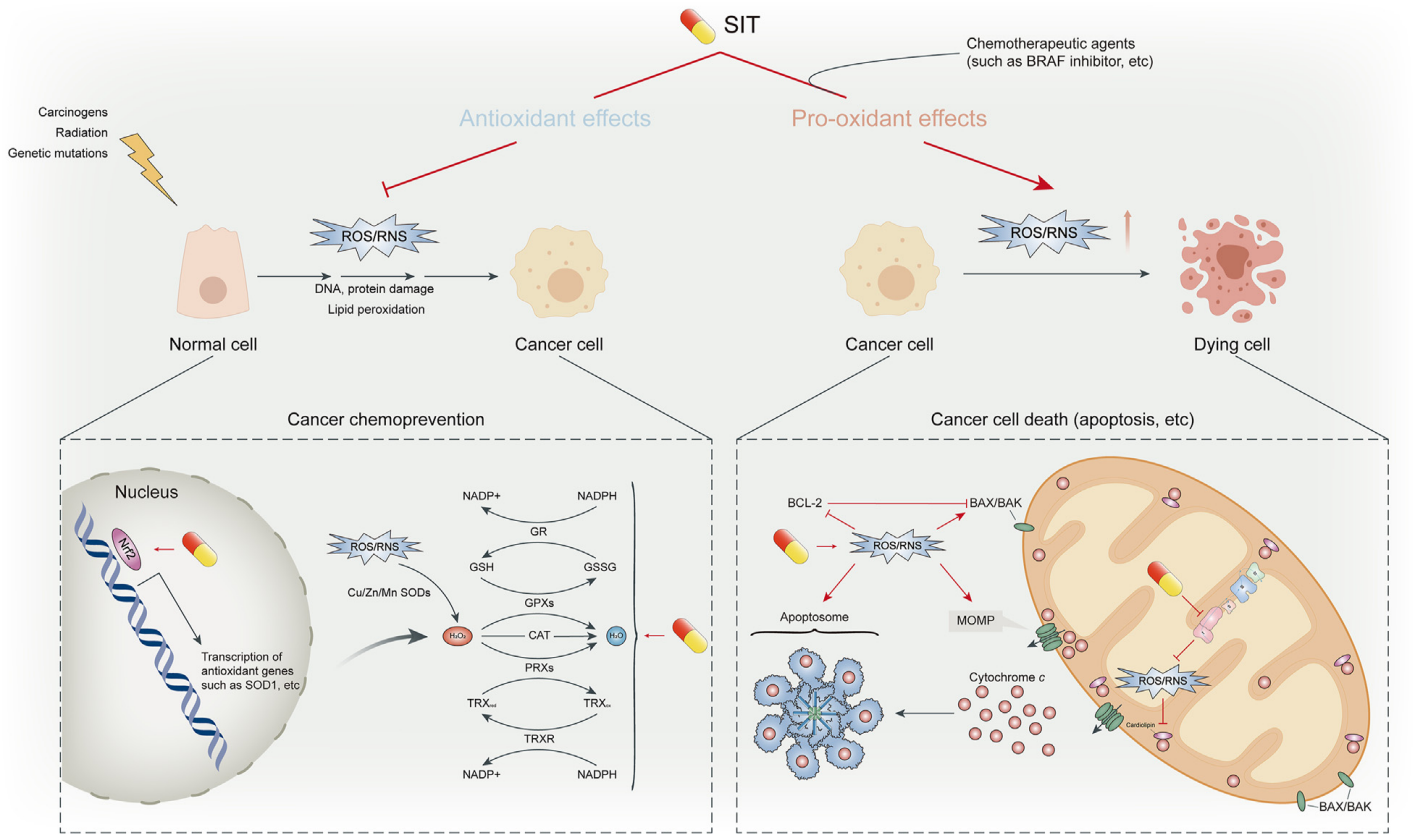
Antioxidant and pro-oxidant effects of β-sitosterol (SIT). The cancer preventive role of SIT is seen in its antioxidant potential, which is able to suppress abnormal ROS/RNS levels in normal cells induced by carcinogens, radiation, genetic mutations, and so on. By scavenging free radicals (not shown) and activating cellular antioxidant defense mechanisms, SIT mitigates oxidative stress and inhibits ROS/RNS-induced macromolecular damage and lipid peroxidation, thereby preventing malignant transformation of normal cells. On the contrary, in the context of cancer treatment, SIT, alone or in combination with chemotherapeutic agents, promotes the excessive accumulation of ROS/RNS by inhibiting ROS/RNS clearance systems and the mitochondrial respiratory chain. SIT-induced excessive oxidative stress causes DNA damage and activates apoptosis-related signals, prompting cancer cell death. The capsule-like symbol represents SIT. ROS, reactive oxygen species; RNS, reactive nitrogen species.

Oxidative stress can activate a variety of transcription factors, leading to the expression of >500 different genes, such as growth factors, inflammatory cytokines, chemokines, cell cycle regulation molecules, and anti-inflammatory molecules [102]. Continued oxidative stress, mainly caused by reactive species such as ROS and reactive nitrogen species (RNS), is closely linked to cancer. SIT, through its antioxidant property, protects cells from undergoing molecular changes that trigger carcinogenesis and plays a key role in cancer chemoprevention; in several instances, owing to its pro-oxidant property, SIT potentiates the efficacy of chemotherapeutic agents by exacerbating oxidative stress, playing a role in cancer chemotherapy (Figure 4).

### Bidirectional regulation of ROS

ROS are highly reactive oxygen–containing molecules, such as hydroxyl (HO∗) and superoxide (O_2_∗) free radicals and nonradical molecules, such as hydrogen peroxide (H_2_O_2_) [103]. It is widely accepted that ROS play a double-edged role in tumor growth, metastasis, and apoptosis, such as ROS-dependent malignant transformation and oxidative stress–induced cell death, which may result from differences in the distributions, concentrations, and durations of ROS in specific subcellular structures [104]. In normal cells, ROS play important roles in homeostasis, and unsurprisingly, ROS concentrations are tightly controlled by cellular antioxidant defense systems to prevent their actions from undesirable consequences [103]. Moderate increases of ROS will contribute to the malignant transformation of normal cells and tumor progression because they initiate pathological transformation of physiological signal networks and induces DNA mutations [105]. Indeed, one important characteristic of cancer cells, compared with their counterpart cells, is the increased ROS concentrations, due to aberrant metabolism, genetic mutations, and relative hypoxia [102]. Then, the wily cancer cells combat the accumulated ROS through elevated concentrations of antioxidants and aberrant nonenzymatic signaling pathways to reinstitute a redox balance and survive in the oxidative stress conditions [102]. However, when ROS accumulation exceeds the critical point, its carcinogenic effects of proliferation and invasion are shifted to antitumor effects. Excessive ROS concentrations damage macromolecular cellular components, such as proteins, lipid bilayers, and chromosomes, resulting in programmed cell death [105]. In general, although abnormal ROS concentrations play contradictory roles in cell growth and death at different stages of cancer formation, both scavenging abnormally elevated ROS to prevent early neoplasia and promoting ROS generation to specifically kill cancer cells are promising anticancer therapeutic strategies [106].

Similar to most phytochemicals, SIT itself exhibits remarkable antioxidant properties. In 2,2'-azinobis(3-ethylbenzothiazoline)-6-sulfonic acid (ABTS) and 2,2-diphenyl-1-picrylhydrazyl (DPPH) assays, SIT was able to trap free radicals in vitro in a dose-dependent manner [41,107,108]. In cultured human colon cancer cell lines, SIT supplementation suppressed the expression of β-catenin and PCNA antigens and reduced the number of aberrant crypt and crypt multiplicity in carcinogen-initiated rats by quenching free radicals [38]. These results suggest that SIT may be capable of preventing mutagenesis and cancer initiation by directly scavenging free radicals. Detoxification of ROS by positively modulating endogenous antioxidants is another mechanism underlying the chemopreventive potential of SIT. Endogenous antioxidants consist mainly of intracellular enzymatic antioxidants, such as superoxide dismutases (SODs), peroxidases (PRXs), glutathione peroxidases (GPXs) and catalase (CAT), in addition to some nonenzymatic antioxidants, such as glutathione (GSH) and NADPH. SODs can rapidly dismutate superoxide into H_2_O_2_, which is then converted into water by PRXs, GPXs, and CAT [[109], [110], [111], [112]]. Oxidized PRXs are reduced by thioredoxin (TRX), which is then restored to the reduced form by thioredoxin reductase (TRXR) [113]. GPXs convert H_2_O_2_ to H_2_O by oxidizing reduced GSH to glutathione disulfide (GSSG), which subsequently undergoes the reduction process catalyzed by glutathione reductase (GR) [114]. Both TRXR-mediated and GR-mediated reduction reactions require NADPH as an electron donor to provide the reducing equivalent. It is also worth mentioning that as the most abundant endogenous antioxidant, GSH can also act as a scavenger to directly react with oxygen-free radicals [115]. Corporately, these endogenous antioxidants sustain the balance between ROS generation and neutralization to protect macromolecules from arbitrary damage caused by oxidative stress, which is of great significance in inhibiting carcinogenesis. Studies have shown that SIT is capable of reinstating and improving cellular antioxidant systems, such as GSH, CAT, SODs, GPXs, GR, and GSH [33,41,116,117]. In particular, through the enhancement of Mn SOD and GPX, SIT alleviated ROS accumulation in phorbol esters (PMA)-treated RAW 264.7 macrophages through the estrogen/PI3K pathway, thereby restoring the impaired GSH/GSSG ratio [118]. As indispensable defensive cells, macrophages often produce and expose to high concentrations of ROS especially in the TME. A common feature of macrophages protecting themselves from ROS damage is the high intracellular GSH content and the rapid turnover of GSH redox cycle [119]. Consequently, the beneficial recovery of GSH/GSSG balance by SIT may contribute to the survival and antitumor effects of macrophages. Similar results were reported in melanoma-bearing mice: SIT significantly enhanced antioxidant activity and proliferation of cytotoxic T cells and macrophages, which may be responsible for inhibiting the expansion of transplantable tumors [120]. The mechanism by which SIT strengthens the endogenous antioxidant system is partly attributable to its activation of the transcription factor Nrf2, which is at the core of a classic antioxidative stress–related pathway contributing to the upregulation of antioxidant defense mechanisms [121,122].

On the contrary, SIT-induced excessive accumulation of intracellular ROS makes great contributions to its antitumor effect [[32], [33], [34],^48^,^49^,^51^]. In these studies, SIT gradually induced ROS accumulation in a concentration-dependent manner. Excess ROS modulates the ratio of 2 antagonistic proteins of the BCL-2 family through DNA damage-induced p53 activation or, otherwise, subsequently enhancing MOMP and activating the intrinsic apoptosis pathway [27]. In addition, caspase 9 and cardiolipin are direct targets of ROS [123,124]. The affinity of cardiolipin for cytochrome c is weakened after oxidation by ROS, which facilitates the release of cytochrome c into the cytosol. Pretreatment with *N*-acetyl-l-cysteine, an exogenous chemical antioxidant, significantly counteracted ROS accumulation and abrogated the antiproliferation and proapoptotic ability of SIT [48,49]. The mechanism underlying SIT-induced excessive ROS accumulation partly lies in SIT’s strong downregulation of TRX1 and TRXR1 proteins [49]. As important molecules involved in ROS clearance, the hyperactivation of TRX1 and TRXR1 has been reported in numerous cancer cells to disturb the redox status, promote cell growth, and encourage apoptotic resistance 113]. Another mechanism to increase oxidative stress is that SIT effectively reduced mitochondrial respiratory capacity, mediated by the inhibition of mitochondrial complex I [51]. This action also makes SIT a promising adjuvant to BRAF inhibitor therapy in patients with melanoma brain metastases. Moreover, SIT stimulates the sphingomyelin (SM) cycle and ceramide (CER) production (discussed later), which have been proved to increase ROS in some cells [125]. Other potential mechanisms merit further investigation.

### Bidirectional regulation of the iNOS/NO/RNS axis

NOis a free radical transmitter that regulates various biological functions in the body. NO can form RNS by interacting with superoxide radicals [126]. Intracellular NO production is catalyzed by the enzyme nitric oxide synthase, which consists of 3 isoforms: neuronal NO synthase, endothelial NO synthase, and inducible NO synthase (iNOS). Among them, iNOS stands apart by the fact that it produces more NO than the other 2 constituent members [127]. Under the regulation of various factors and pathways, iNOS expression levels and NO content are often abnormally altered in tumors, particularly modulating important tumor-related processes [128]. Similar to ROS, it has been widely acknowledged that iNOS-derived NO and RNS have the disputed dual role of tumorigenic or tumoricidal activities [129]. Moderate concentrations of NO/RNS are believed to promote cell proliferation and increase genomic instability, whereas preventing apoptosis and impairing DNA repair, thus conducive to tumorigenesis. However, high concentrations of NO/RNS, produced by the tumor cell itself or originated from immune cells infiltrated in the TME, can exhibit proapoptotic, migration-inhibitive, cytotoxic, and antitumoral properties.

There have been reports that, even in small amounts, SIT has an effect on the NO signaling pathway. SIT reduced PMA-induced NO synthesis in RAW 264.7 macrophages in a concentration-dependent manner, which was due to the reversal of elevated iNOS expression levels [130]. During early events of tumorigenesis, macrophages produce high concentrations of NO/RNS to initiate apoptosis of tumor cells [131]. Therefore, the action of SIT on the carcinogen PMA-treated macrophages seems to be adverse from an antitumor perspective. By contrast, Boubaker et al. [120] found that peritoneal macrophages isolated from SIT-treated melanoma-bearing mice showed a significant increase in NO production. The enhanced NO synthesis reflects phagocytic stimulation, which may help kill tumor cells. In another study, SIT markedly downregulated the mRNA and protein concentrations of iNOS in the esophagus of rats treated with NMBA, an esophageal carcinogen. The concentrations of nitrite in the esophagus decreased correspondingly [132]. Given that nitrite is a strong carcinogenic factor for esophageal cancer, SIT played a protective role in this context. Overall, considering a cancer cell, recent advances argue that the cellular outcome in the face of NO/RNS has to be interpreted regarding additional factors of response, such as the tumor type, duration or timing of NO/RNS delivery, and TME [129]. Therefore, either promotion or inhibition of the iNOS/NO/RNS axis by SIT may have anticancer potential. However, comprehensive consideration and further research are still needed.

### Inhibition of lipid peroxidation

Lipids are essential components of cell membranes that maintain cell structure and control the cell function. They are prime targets for reactive species attacks. High levels of oxidative stress cause lipid peroxidation, producing peroxides such as reactive lipid species. Lipid peroxidation is believed to be mutagenic and carcinogenic and has been intensively implicated in cancer [133]. SIT is able to mitigate lipid peroxidation, thereby eliminating the tumor-promoting effects of lipid peroxidation.

Malondialdehyde (MDA) is an indicator of membrane lipid peroxidation, representing the intense attack at polyunsaturated fats and membrane degradation. SIT reduced the concentrations of MDA in the hepatocytes, kidney, and serum of melanoma-bearing mice [120]. Similarly, in 1,2-dimethylhydrazine (DMH)-induced rats with colon carcinogenesis, elevated concentrations of liver lipid peroxides by DMH induction were effectively reversed with SIT supplementation. In line with that, DMH-induced histopathologic alterations were restored to near normal conditions [117]. LDL is susceptible to lipid peroxidation. Peroxidized LDL can be taken up by CD8^+^ tumor–infiltrating lymphocytes, causing dysfunction of CD8^+^ tumor–infiltrating lymphocytes [134]. Studies have reported that SIT could protect the physicochemical properties (fluidity and molecular order) and conformation of LDL from lipid peroxidation. In participants with SIT supplementation in their diet, a remarkable reduction of oxidized LDL and a slight reduction of plasma isoprostanes (a marker of free radical–induced lipid peroxidation) were observed [135,136]. Finally, SIT was able to decrease the degree of lipid peroxidation in platelet membrane at low concentrations [137].

However, the existing research results have confirmed only that SIT has an inhibitory effect on lipid peroxidation, which is consistent with the role of lipid peroxidation in cancer occurrence and development. No evidence shows that SIT promotes lipid peroxidation or that its antitumor effects arise from the enhancement of lipid peroxidation. Therefore, future research could continue to explore this possibility.

## Effects on metabolic reprogramming

**FIGURE 5:**
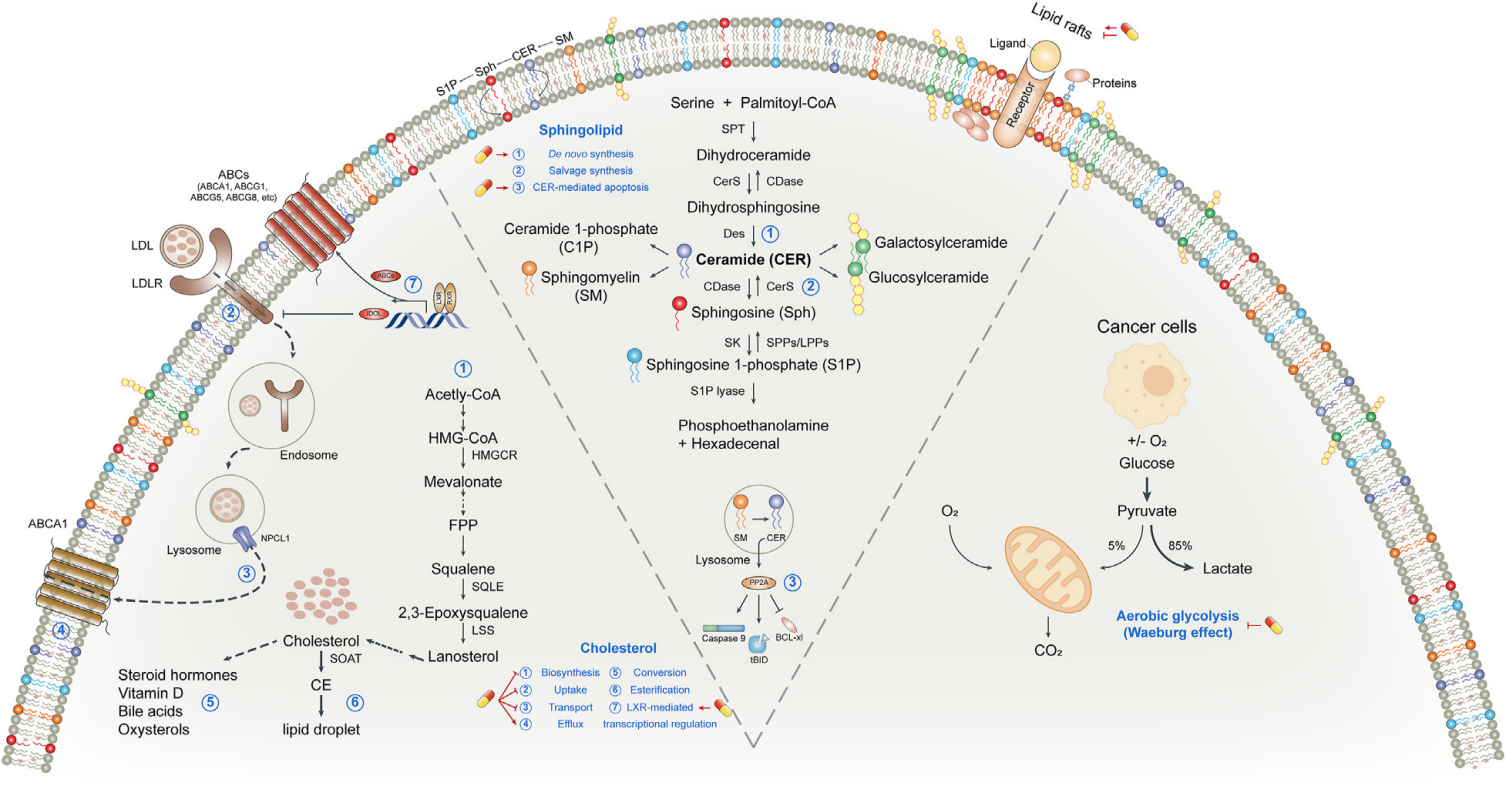
Regulation of metabolic reprogramming and cell membrane structure. Left panel (cholesterol metabolism): β-sitosterol (SIT) inhibits de novo cholesterol biosynthesis (①) by inhibiting HMGCR or other enzymes in the mevalonate pathway. SIT inhibits LDLR-mediated cholesterol uptake by directly downregulating membrane LDLR expression (②) or by activating LXRs to promote IDOL transcription (⑦). SIT decreases the expression of NPCL1 transporter (③) which is responsible for cholesterol transport. As an agonist of LXRs, SIT promotes the expression of LXR target genes ABC transporters (⑦), thereby increasing cholesterol efflux (④). In addition, SIT has a weak estrogenic effect (⑤). SIT has no influence on the cholesterol esterification process (⑥). Middle panel (sphingolipid metabolism): SIT promotes de novo CER synthesis by increasing the activity of SPT (①). The influence of SIT on CER catabolism and the salvage synthesis pathway is unclear (②). SIT induces an increase in CER production and activates PP2A, which promotes apoptosis by regulating molecules in the apoptosis pathway (③). In particular, SIT alters intracellular levels of sphingolipids and cholesterol, which are essential components of membrane lipid rafts. Therefore, receptors and signaling pathways associated with membrane lipid rafts may be significantly affected by SIT. Right panel (glucose metabolism): Although when oxygen is plentiful, tumor cells rely heavily on glycolysis to produce energy, a phenomenon known as the “Warburg effect.” SIT has an antiglycolytic effect on cancer cells. The capsule-like symbol represents SIT. ABC, ATP-binding cassette; CER, ceremide; HMGCR, 3-hydroxy-3-methylglutaryl-CoA reductase; IDOL, inducible degrader of the LDLR; LDLR, LDL receptor; LXR, liver X receptor; NPCL1, Niemann-Pick C1-like 1; PP2A, protein phosphatase 2A; SPT, serine palmitoyltransferase.

Metabolic reprogramming is a hallmark of cancer, enabling tumor cells to meet the increased energy demands required for rapid proliferation, invasion, and metastasis [138]. Elucidating the underlying mechanisms of cancer metabolic reprogramming will help identify cancer targets and therapeutic strategies. Current studies suggest that improving tumor metabolic reprogramming, primarily lipid (such as cholesterol and sphingolipids) and glucose metabolism, thereby limiting the access to nutrients and remodeling membrane structure, is an important part of SIT’s antitumor mechanisms (Figure 5).

### Regulation of cholesterol metabolism

Cholesterol is an important component of cell membranes and has several functions, such as controlling membrane fluidity, regulating the activity of membrane binding proteins, and coordinating multiple signaling pathways, in addition to immune responses [139]. Cholesterol is essential for the growth and metastasis of cancer cells. Targeting cholesterol metabolism, such as blocking its synthesis or uptake, could provide a therapeutic window for cancer treatment [140].

Indeed, the well-recognized clinical outcome of SIT intake is the hypocholesterolemic effect. Numerous trials have demonstrated that SIT consistently and dose dependently reduce serum cholesterol concentrations in various populations and patient groups and in mice [[141], [142], [143]]. The commonly accepted basic mechanism of action is that SIT and cholesterol have similar structures and differ only in 1 ethyl group at the C-24 site; so, SIT can competitively block cholesterol absorption from the intestinal lumen [144]. SIT is also more hydrophobic than cholesterol and has greater affinity for micelles, which allows it to effectively bind to micelles in the intestinal cavity and replace cholesterol [145]. In addition, in cultured enterocytes, SIT has been shown to decrease the expression of Niemann-Pick C1-like 1 transporter, which is responsible for cholesterol absorption [146].

At the cellular level, SIT supplementation has been observed to cause a reduction in intracellular cholesterol content of tumor cells [85]. In particular, SIT reduces cholesterol concentrations in tumor cell membranes. SIT treatment in colon cancer cells resulted in a 26% reduction in membrane cholesterol [147]. Cholesterol is a well-known membrane microdomain stabilizer. The reduction of membrane cholesterol weakens membrane fluidity, therefore attenuating the adhesion, motility, and aggressiveness of tumor cells [148]. Similarly, supplementation of SIT in breast cancer cells led to enrichment of SIT in cell membranes and caused a decrease in the membrane cholesterol content by ∼50%, accompanied by a significant increase in the concentrations of Fas receptors and inhibition of tumor cell growth [66]. Fas receptors have been reported to localize in cholesterol-rich membrane lipid rafts [149]. It is reasonable to assume that SIT disrupts the structure and stability of lipid rafts by altering membrane cholesterol content, thereby affecting the signal transduction through Fas receptors which leads to tumor cell apoptosis. Moreover, Lomenick et al. [150] found that SIT had a high affinity for extended synaptotagmin (E-Syt)1. E-Syt is located in the endoplasmic reticulum, where it tethers the endoplasmic reticulum membrane with the plasma membrane. Its isomer E-Syt2 functions in the lipid transfer between endoplasmic reticulum and plasma membrane. It is possible that, by binding E-Syt, SIT interferes with the dynamic transport of cholesterol to the destined membrane for structural and functional needs. Collectively, SIT changes membrane structure through its cholesterol-lowering effects, which may induce alteration in signal transduction affecting cell growth in tumor cells.

The mechanism by which SIT lowers cholesterol content in tumor cells is, on the one hand, inhibiting cholesterol biosynthesis. Every mammalian cell can synthesize cholesterol through the mevalonate pathway [139]. Three acetyl-CoA molecules in the cytosol condense to form 3-hydroxy-3-methylglutaryl-CoA, which is then reduced to mevalonate by 3-hydroxy-3-methylglutaryl-CoA reductase (HMGCR), the major rate-limiting enzyme in cholesterol biosynthesis. A series of enzymatic reactions convert mevalonate to farnesyl pyrophosphate (FPP) and the condensation of 2 FPP molecules into squalene commits the process to sterol production. Squalene is subsequently oxidized and cyclized to lanosterol, which follows the Bloch pathway, the Kandutsch–Russell pathway, or a hybrid pathway before it is ultimately converted to cholesterol. SIT effectively impedes cholesterol biosynthesis in different cancer cell lines, such as breast [151] and colorectal cancers [152]. In the former, exogenous mevalonate failed to reverse this inhibition, indicating that the regulated target was not HMGCR but downstream from the rate-limiting step of de novo cholesterol synthesis. However, in the latter, protein and mRNA concentrations of HMGCR decreased under SIT treatment. These results indicate that SIT has different mechanisms of action to inhibit cholesterol biosynthesis in different cancer types. The portion of biosynthesized cholesterol in excess of current cellular demands is converted to cholesteryl ester by acyl-coenzyme A: cholesterol acyltransferases (ACATs, also known as SOATs) [153]. The study in colorectal cancer found that cholesterol esterification and acyl-coenzyme A: cholesterol acyltransferase activities were uninfluenced with SIT treatment [152], but the case in other cancer types is not investigated.

To the contrary, SIT lowers cholesterol concentrations in tumor cells by interfering with cholesterol uptake. Compared with colorectal cancer cells incubated with micelles containing cholesterol, those incubated with SIT in the micelle together with cholesterol interfered with the uptake of micellar cholesterol, weakening the influx of plasma membrane cholesterol [152]. Most mammalian cells, such as cancer cells, obtain cholesterol from LDL in blood circulation through LDL receptors (LDLR)-mediated endocytosis. After the binding of LDL to LDLR, the complex is internalized into the cell endosome. In the endosome, LDL is dissociated from LDLR and further transferred to the lysosome, where the free cholesterol is released [154]. Some tumors prefer to use exogenous cholesterol at the expense of the more time-consuming and energy-consuming de novo cholesterol synthesis [155,156], whereas other tumors rely entirely on LDLR-mediated exogenous cholesterol uptake due to defects in the cholesterol biosynthesis pathway [157], highlighting the importance of exogenous cholesterol uptake for tumor cell survival. Correspondingly, tumor cells promote the expression of LDLR to acquire abundant exogenous cholesterol. Blocking LDLR-mediated cholesterol uptake is a promising therapeutic approach and has been shown to effectively promote tumor cell death in glioblastoma [158] and pancreatic cancer [156]. The LDLR expression in hepatoma cells decreased significantly after exposure to *Girardiana diversifolia* extracts for 72 h, among which SIT was the most abundant component [159]. The extracts also had a significant cytotoxic effect on tumor cells, implying that SIT may inhibit tumor growth by downregulating LDLR to cause a profound change in intracellular cholesterol homeostasis. In this context, SIT provides an additional option for anticancer drugs targeting LDLR.

The organic orchestration of cholesterol biosynthesis, uptake, efflux, and esterification contributes to cholesterol homeostasis, which is subject to stringent and fine-tuned regulations. The master transcriptional regulator that governs cholesterol homeostasis and has been recognized as the target of SIT is liver X receptors (LXRs). LXRs are ligand-activated transcription factors with 2 isoforms (LXR-α and LXR-β) belonged to the nuclear receptor superfamily [160]. In response to high cellular cholesterol concentrations, desmosterol (the immediate precursor of cholesterol) and oxysterols bind and activate LXRs, thereby enhancing the expression of genes associated with cholesterol efflux, such as ATP-binding cassette subfamily A member 1 (*ABCA1*) and ATP-binding cassette subfamily G member 1 (*ABCG1*) [161], genes associated with cholesterol exertion, such as *ABCG5* and *ABCG8* [162], and others, such as inducible degrader of the LDLR (also known as *MYLIP*) [163], to promote elimination of excess cholesterol. LXRs agonists have shown promising efficacy in the treatment of several cancers, mainly by inhibiting cancer cell proliferation and inducing apoptosis [164]. For example, LXRs activation affects multiple regulators of the cell cycle through lipogenic activity, ultimately leading to cell cycle arrest at the G1 phase [165]. Activating LXRs signaling also promotes T cell activation that could augment other immunotherapies [166]. With the LXRs coactivator peptide recruitment assay, SIT was confirmed to activate LXR-α and LXR-β [167]. The functionality of this effect was further demonstrated in Caco-2 cells by increased expression of *ABCA1*, one of the known LXRs target genes [167]. Similarly, mRNA concentrations of LXR-α and LXR-β were significantly upregulated in colon adenocarcinoma cells after 48 h of SIT treatment [168]. Of particular importance, most LXRs agonists have a regulatory effect not only on cholesterol metabolism but also on hepatic expression of LXR target genes involved in fatty acid metabolism. This would lead to unwanted hypertriglyceridemic effects during LXRs agonist treatment [160]. However, as an effective LXRs agonist, SIT does not adversely affect hepatic triglyceride metabolism [169]. These evidences indicate that SIT can be used as a safe and effective LXRs agonist for cancer treatment.

More than just a membrane component, cholesterol is a precursor to steroid hormones that can trigger or promote breast and prostate cancers [170]. Shown in an array of in vitro test systems, SIT had weak estrogenic potency, ∼1/1000 to 1/10,000 of the potency of E_2_ and had almost equal binding affinities for estrogen receptor (ER) subtypes ER-α and ER-β [171]. However, the effect of SIT as a potential ER agonist on tumors has not been adequately studied. It has been reported that intake of SIT is associated with a greater likelihood of ER-positive (ER+) tumors than ER-negative (ER−) ones (OR: 0.42; 95% CI: 0.18–0.98) [172]. Moreover, unlike its proapoptotic effects in the estrogen-independent breast cancer line MDA-MB-231, SIT (>1 μM) promoted the growth of estrogen-dependent breast cancer cell line MCF-7 in vitro [173]*.* Nevertheless, in vivo experiments in the same study exhibited the opposite results: not only did SIT (9.8 g/kg diet) not stimulate MCF-7 tumor growth in ovariectomized athymic mice but significantly reduced E_2_-induced tumor growth and lowered serum E_2_ concentrations [173]. The reason of these results is not yet known. It is hypothesized that SIT can act as an estrogen analog to promote the growth of hormone-dependent tumors when acting alone, whereas in the presence of high-potency estrogen, SIT may competitively block its tumor-promoting effect.

### Regulation of sphingolipids metabolism

Sphingolipids are structural molecules of cell membranes, which play an important role in maintaining barrier function and fluidity [174]. Sphingolipids also regulate various biological processes such as growth, proliferation, migration, and invasion by modulating signal transduction networks of cancer cells [175,176]. Multiple signaling nodes in sphingolipids metabolism provide new therapeutic targets for the development of novel anticancer strategies and are closely related to the pharmacologic effects of SIT.

At the center of the sphingolipids metabolic pathway is proapoptotic CER, which can be synthesized de novo with serine and palmitoyl-CoA as raw materials successively catalyzed by serine palmitoyltransferase, ceramide synthetases 1–6, and dihydroceramide desaturase. CER can be reversibly interconverted into SM, glucosyl/lactosyl/galactosyl ceramides and other complex glycosphingolipids, or phosphorylated into prosurvival ceramide 1-phosphate. Catabolism of CER produces another proapoptotic sphingolipid, sphingosine, which can be converted into prosurvival sphingosine 1-phosphate (S1P) by the action of sphingosine kinases 1/2. Ultimately, S1P can be either recovered into sphingosine by the action of S1P phosphatases or lipid phosphate phosphatases, initiating the salvage synthesis pathway of CER, or irreversibly degraded by S1P lyase to produce proapoptotic hexadecenal and phosphoethanolamine phosphate [174].

In breast cancer, SIT treatment increased CER production in MCF-7 and MDA-MB-231 cells [177]. The mechanism was that SIT increased the activity of serine palmitoyltransferase, the rate-limiting enzyme for de novo CER synthesis. When used in combination with tamoxifen in MDA-MB-231 cells, SIT further enhanced the promoting effect of tamoxifen on CER synthesis [177]. Similarly, SIT-induced increase in CER production were observed in colon [178] and prostate cancers [179]. In HT-29 and LNCaP cells, supplementation of SIT at 16 mM for 5 and 7 d increased CER production by 45% and 50%–55%, respectively. Because the addition of SIT had no effect on sphingosine content [178], it can be inferred that SIT may not promote the production of CER through the salvage pathway. However, it still remains to be further investigated that which targets in the sphingolipids metabolic network are affected by SIT in different cancer types. In parallel, SIT-induced increased CER is accompanied by inhibition of cancer cell growth and enhancement of apoptosis. The intermediate bridge linking the increased CER and tumor cell apoptosis is most likely protein phosphatase (PP)2A. As shown by Awad et al. [180], although SIT did not alter PP2A protein concentrations, it increased PP2A activity by 50% in LNCaP cells. PP2A is a major serine/threonine phosphatase that exerts tumor suppressive activity by targeting proliferative kinases, cell cycle regulators, and apoptosis inhibitors and has been reported to be a direct target for CER action on cell growth and apoptosis [181]. Therefore, activating PP2A through improving CER production is undoubtedly an indispensable part of the anticancer mechanisms of SIT.

The influence of SIT on sphingolipids metabolism occurs not only in the cytoplasm but also in the membrane of cancer cells. HT-29 cells supplemented with SIT had decreased membrane SM and increased membrane CER, whereas the growth was only one third of the cells added with equimolar concentration of cholesterol [147]. CER has been shown to diminish cholesterol content of membrane lipid rafts. Once the composition of lipid rafts such as sphingolipids or cholesterol is altered, the associated signaling moieties are profoundly influenced in sequence [182]. A strong piece of evidence is that incorporation of SIT into liver microsome membranes decreased membrane fluidity, further influencing the activity of 3 integral enzymes (A5, A6, and A9 desaturase) [183]. The significance of these functional alterations in tumor development has not been investigated. In addition, in 3 separate studies, HąC-Wydro et al. used 3 kinds of Langmuir monolayer to mimic the tumor cell membrane to explore how SIT affected membrane structure and properties. It was found that SIT supplementation reduced cholesterol concentrations, lowered SM concentrations, and increased CER concentrations, leading to the weakness of the film’s condensation and stability [184]. Membranes containing a higher proportion of unsaturated phosphatidylcholine [185] or choline plasmalogens [186] (e.g., breast or colon cancer cell membranes) were more susceptible to SIT. From this perspective, as a strong membrane-modifying compound with influence on sphingolipids metabolism, SIT can combat cancer by interfering with the physicochemical properties of membranes and membrane protein–mediated signal transduction.

### Regulation of glucose metabolism

Reprogramming toward high glucose metabolism has been considered one of the hallmarks of cancer, which is mediated by oncogenic drivers and by the undifferentiated character of cancer cells and is necessary for cancer cells to meet high anabolic demands [187]. Several preclinical and early-stage clinical studies have shown that interventions aimed at modulating glucose signaling may prove salutary in cancer therapy [187]. An important aspect of the reprogrammed glucose metabolism is that tumor cells rely heavily on glycolysis to produce energy, even in the presence of sufficient levels of oxygen, a phenomenon known as the “Warburg effect” [188]. A recent study demonstrated that SIT had an antiglycolytic effect on cancer cells: the percentage of glucose uptake and lactate concentration in SIT-treated MDA-MB-231 cells were significantly lower than those in untreated cells in a time-dependent manner [189]. Thus, SIT may be more effective if used as a metabolic antiglycolytic agent in drug combinations to treat cancer. Cancer cells adapt to the low energy yield of glycolysis by increasing glucose uptake to support a higher glycolysis rate. Hence, the strategy of starvation therapy through depleting glucose and other critical nutrients of tumors has been widely studied as an attractive form of cancer treatment [190]. The hypoglycemic effect of SIT has been observed in several studies [159,191,192]. Mechanically speaking, on the one hand, SIT had moderate inhibitory activity on 2 enzymes involved in carbohydrate digestion, α-amylase and α-glucosidase [159]. To the contrary, SIT was able to increase the amount of insulin and improve insulin resistance in insulin-dependent tissues [[191], [192], [193]]. Insulin resistance has been linked with an increased incidence of various cancers through mechanisms such as upregulating *IGF-1*, increasing the concentrations of bioavailable estrogen, and altering the inflammatory cytokine profile [194]. Jointly, given the association between glucose intake and cancer development, SIT may be of benefit in slowing tumor progression. However, further studies are required to confirm a direct relationship between SIT’s hypoglycemic function and its anticancer effects.

## Effects on tumor metastasis

**FIGURE 6:**
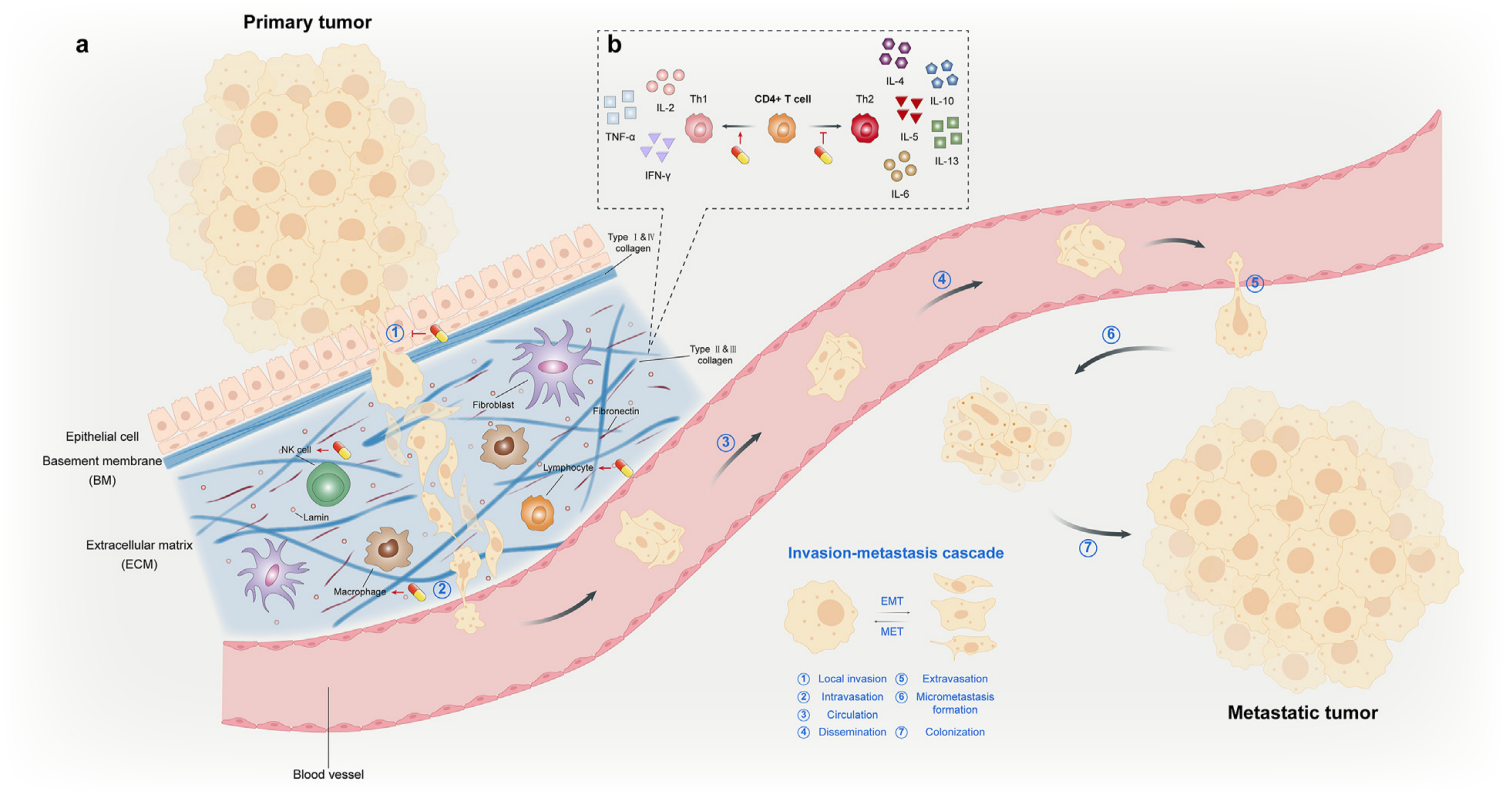
Invasion–metastasis cascade and tumor microenvironment. The invasion–metastasis cascade is a series of biological events that occur during tumor metastasis, such as the departure from primary growth sites, translocation in the systemic circulation, and survival and colonization in distant tissues. The invasion–metastasis cascade can be subdivided into 7 processes. Meanwhile, EMT and its counter-process MET play an important role in tumor metastasis. On the one hand, β-sitosterol (SIT) prevents tumor cells from acquiring a mesenchymal phenotype and detaching from surrounding cells by inhibiting EMT. To the contrary, SIT inhibits the erosion of the BM and ECM by tumor cells. Taken together, SIT mainly blocks the invasion–metastasis cascade in the initial process (part **a**). Whether during metastasis or at primary/distant colonization sites, immune cells are an important component of the TME and play a tumor-suppressing or tumor-promoting role. SIT promotes lymphocyte proliferation and strengthens the activity of a variety of immune cells, such as NK cells and macrophages. The regulation of immune cells by SIT enhances the effect of killing tumors and inhibiting metastasis. In addition, SIT is able to shift the T_H_1/T_H_2 balance toward T_H_1, which is abnormally inclined toward T_H_2 in the TME. Thus, antitumor effects of T_H_1 cytokines are enhanced and tumor-promoting effects of T_H_2 cytokines weakened (part **b**). The capsule-like symbol represents SIT. BM, basement membrane; ECM, extracellular matrix; EMT, epithelial-mesenchymal transition; MET, mesenchymal–epithelial transition; T_H_, T-helper subset; TME, tumor microenvironment.

Although surgical resection and adjuvant therapy can cure well-localized primary tumors, metastatic tumors are essentially incurable owing to their systemic nature and resistance to existing therapeutic agents. In reality, >90% of clinical mortality from cancer is attributable to metastases rather than the primary tumors [195]. Therefore, our ability to effectively treat cancer depends to a large extent on our capacity to block and perhaps even reverse the process of metastasis. Tumor metastases are the culmination of a complex series of cell-biological events collectively known as the invasion–metastasis cascade (Figure 6). During metastatic progression, tumor cells leave their primary sites of growth (local invasion and intravasation), translocate systematically (survive in the transport through the vasculature, arrest at distant organ sites, and extravasation), and adapt to survive and thrive in these foreign microenvironments of distant tissues (micrometastasis formation and metastatic colonization) [196].

SIT impedes the invasion–metastasis cascade mainly by interdicting the beginning local invasion process. Local invasiveness involves entry of cancer cells within the primary tumor into the surrounding tumor-associated stroma and thereafter into the adjacent normal tissue parenchyma. To invade the stroma, incipient metastatic cancer cells must first destroy the basement membrane (BM), a special type of extracellular matrix that plays a crucial role in organizing epithelial tissues and acts as an intrinsic barrier to local invasion [197]. In addition to its structural role, the BM contains a repository of growth factors that can be liberated by carcinoma-secreted proteases. The BM also plays key roles in signal transduction events in cancer cells through pathways initiated by integrin-mediated cell–matrix adhesion, leading to alterations in cell polarity, proliferation, and invasiveness [198]. In vitro SIT supplementation significantly inhibited the binding of prostate cancer cells to laminin and fibronectin, 2 important BM components, and attenuated the invasion and migration of tumor cells as shown in the transwell assay. In parallel, the number of mice with lymph node and lung metastases in the SIT-fed group was approximately half that of the control group [199]. The suppressive effect of SIT on adhesion of tumor cells to several BM proteins (collagen I, collagen IV, fibronectin, and laminin) has also been demonstrated in breast cancer [85,200]. These effects may arise from SIT affecting the expression of integrin receptors on the surface of tumor cells. Concomitantly, mice fed SIT showed 33% smaller breast tumors and 20% lower metastasis than those fed cholesterol [200].

In addition to the obstruction of the well-structured BM, E-cadherin–mediated tight intracellular junctions also prevent dissociation of individual epithelial cells from their primary site. To overcome this obstacle, cancer cells may opt for a developmental program termed epithelial-mesenchymal transition (EMT), which is critical for embryogenesis and wound healing. During EMT, adherent epithelial cells shed connections with neighboring cells, change their polarity and acquire the characteristics of migratory mesenchymal cells, thus taking on enhanced motility [201]. EMT programs are orchestrated by a group of pleiotropically acting EMT-transcription factors, such as Slug, Snail, Twist, ZEB1, and ZEB2, which promote entrance into a mesenchymal state by inhibiting expression of epithelial markers and inducing expression of other mesenchymal markers [202]. In addition, epigenetic and posttranslational modifications are involved in the regulation of EMT. For example, DNA methyltransferase (DNMT) 1-mediated DNA methylation and histone deacetylase (HDAC) 1/2–mediated histones modification are mechanisms of E-cadherin inhibition [203,204]. Epithelial-originated tumor cells can take advantage of EMT to achieve the first steps of the invasion–metastasis cascade, such as local invasion into the stroma, intravasation, and possibly extravasation at distant areas. Then, the reverse process, mesenchymal–epithelial transition helps mesenchymal tumor cells that reach the metastatic site regain adhesion and successfully form secondary tumors [201]. By reshaping EMT, tumor cells can obtain increased motility/aggressiveness, stem cell properties, and drug resistance, so it is no surprise that EMT has become an attractive target for cancer therapy [202]. SIT significantly attenuated EMT in pancreatic cancer cells by dose dependently downregulating Snail and vimentin, wheras upregulating E-cadherin. Consistently, migration and invasion of the pancreatic cancer cells were inhibited [53]. This inhibitory effect of EMT was accompanied by a dose-dependent decrease in phospho-AKT and phospho-GSK-3β concentrations and further enhanced by the combined treatment with PER, the AKT inhibitor, or LiCL, the GSK-3β inhibitor, suggesting that the AKT/GSK-3β pathway is involved in the inhibition of EMT by SIT [53]. In addition, SIT can interfere with the epigenetic modifications in favor of EMT. Sublethal dosage of H_2_O_2_ distinctly decreased the expression of *CDH1* (the coding gene of E-cadherin) at the mRNA and protein concentrations, promoting the migration of breast cancer cells. The upregulation of DNMT1 and HDAC1 and the activation of ERK/Snail/Slug axis indicated that DNA methylation, histone modification, and transcriptional regulations were jointly involved in the ROS-mediated EMT facilitation. However, fluorescence measurement using DCFDA dye confirmed that H_2_O_2_-induced ROS concentrations in tumor cells were significantly reduced in the presence of SIT, and the application of SIT as a ROS scavenger remarkably attenuated the promoting effect of ROS-induced epigenetic modifications on EMT [205]. Similarly, SIT inhibited the growth and migration of prostate cancer cells, which was associated with upregulation of the tumor suppressor gene *CDH1* and downregulation of DNMT1 and HDACs, suggesting that SIT can target epigenetic regulatory mechanisms involved in EMT [54].

Once metastatic cancer cells have broken away from neighbors and broken through the BM, they enter the stroma where they are confronted with a variety of tumor-associated stromal cells such as fibroblasts, adipocytes, and macrophages and other immune cells. Bidirectional interactions will occur between them: carcinoma cells stimulate the transformation of stromal cells into a protumorigenic phenotype, and these stromal cells further enhance the aggressive behaviors of carcinoma cells through various types of heterotypic signaling [206]. Reeducating stromal cells and blocking this self-amplifying positive feedback loop seems to be an effective strategy for treating cancer. After daily oral administration of liposomal SIT for 7 d, NK cell activity and the amount of immune response cytokines increased, whereas the number of pulmonary melanoma metastatic colonies was significantly lower than that in the control group [207]. Similarly, SIT significantly enhanced the activity of macrophages and cytotoxic T cells in melanoma-bearing mice and remarkably impeded the expansion of transplantable tumors into the lung parenchyma [120]. However, whether these affected immune cells are derived from the tumor stroma remains unknown. Further studies are needed to prove causality between SIT’s remodeling of stromal cell activity and suppression of local invasion.

In general, SIT inhibits multiple biological processes associated with local invasion; however, the effect of SIT on other steps of the invasion–metastasis cascade needs further study. In addition, the role of EMT in tumor metastasis has been heatedly debated with recent reports both refuting and supporting the requirement for EMT [201], which adds to the complexity of SIT’s mechanism of action on tumor metastasis.

## Effects on immunity and inflammation

The activity and composition of immune cells and the secretion of cytokines largely determine the normal function of the immune system, which has a pivotal role in cancer prevention and prognosis. Inflammation is an inducer of carcinogenesis, whereas the inflammatory TME is believed to be a promoter for cancer development and invasion [208]. The proinflammatory mediator cyclooxygenase (COX)-2 is essential for the formation and maintenance of a tumorigenic inflammatory TME. SIT has immunomodulatory effects on immune cell activity, composition, and COX-2–mediated inflammatory response.

### Modulation of the T_H_1/T_H_2 imbalance

One preliminary study showed that SIT and its glycoside (SITG) enhanced the proliferation of mitogens-activated human peripheral blood lymphocytes both in vivo and in vitro, and the mixture of the 2 exhibited a more powerful stimulatory effect [209]. In melanoma-bearing mice, the tumor significantly inhibited splenocytes proliferation, impaired cytotoxic ability of T lymphocytes, and weakened macrophage lysosomal activity, whereas SIT significantly restored these immune functions. These immune-boosting effects of SIT remarkably inhibited lung metastasis of transplanted melanoma [120]. Similarly, liposomal delivery of SIT in a murine model of melanoma improved gut immune surveillance system manifested by increased immune response cytokines and NK cell activity, thereby attenuating tumor metastasis [207]. Thus, stimulatory effects of SIT on immune cell activity may be a means by which it exerts chemopreventive and chemotherapeutic effects.

T cells consist of 2 distinct subpopulations, CD4^+^ helper cells and CD8^+^ cytotoxic cells. Depending on the type of invading pathogen, CD4^+^ T cells develop into different T-helper (T_H_) subsets, T_H_1 and T_H_2, characterized by specific cytokine patterns secreted on stimulation. Type T_H_1 releases IL-2, TNF-α and IFN-γ, wheras type T_H_2 secretes IL-4, IL-5, IL-6, IL-10, and IL-13 [210]. In general, cytokines secreted by T_H_1 cells act as suppressors against a tumor-promoting microenvironment. IFN-γ has an antiangiogenic function in the TME, preventing infiltration and metastasis of tumor cells [211]. IL-2 binds to IL-2 immunoreceptors on the surface of NK cells and enhances the overexpression of cyclin B1, leading to selective NK cell proliferation [212]. As previously mentioned, binding of TNF-α to its receptor triggers cell apoptosis through the extrinsic apoptosis pathway. In addition, TNF-α is able to disrupt the tumor vasculature [213]. On the contrary, cytokines secreted by T_H_2 cells in the TME exert immunosuppressive effects and promote immune evasion. For example, IL-4 binds to IL-4 receptor to phosphorylate STAT6, thereby increasing apoptotic resistance and colonization of tumor cells [214]. IL-13 suppresses the proliferation of CD8^+^ T cells, markedly decreasing the cytotoxic effects on tumor cells [215]. Ideally, a healthy immune system maintains a delicate balance between T_H_1 and T_H_2 cells. However, alterations in cell polarization and cytokine balance, referred to as the T_H_1/T_H_2 shift, have been implicated in malignant tumors. Impaired T_H_1 response and T_H_2 overactivation play an important role in tumor initiation and development [216]. By analyzing the cytokine secretion profile, numerous studies have demonstrated that SIT can cause a shift in the T_H_1/T_H_2 balance toward T_H_1 (Figure 6). Reported by Le et al. [217], SIT blocked the secretion of IL-4 and IL-10 but had no effect on the secretion of IL-2 and IFN-γ in murine T cells, suggesting that SIT selectively inhibited T_H_2 activity and promoted a T_H_1 bias. In another murine model fed SIT, the secretion of IL-2 and IFN-γ increased, whereas the secretion of IL-4 and IL-10 was unchanged, and the T_H_1/T_H_2 ratio upregulated significantly [218]. Similar to the results in mice, the addition of SIT and SITG to human peripheral blood lymphocytes cultured in vitro remarkably increased the secretion of T_H_1 cytokines (IL-2 and IFN-γ) in the medium, which in turn greatly enhanced the lytic/cytotoxic activity of NK cells against cancer cell lines [209]. On the contrary, based on a previous study in the leukemia cell line, SIT did not affect IL-4, IL-10, and IFN-γ secretions but suppressed IL-2 expression in a dose-dependent manner [219]. Although the specific cytokines affected by SIT varied across studies, probably owing to differences in models or other influencing factors, the conclusion that SIT promotes a T_H_1 shift is consistent. However, the underlying mechanism is not fully elucidated. Observed by Brüll et al. [220], administration of SIT to human peripheral blood mononuclear cell increased IL-2 and IFN-γ, whereas slightly decreased IL-4 and IL-10 concentrations. Blocking or knocking out Toll-like receptors 2 reversed this effect, indicating that Toll-like receptors 2 is essential in the SIT-mediated T_H_1 shift. Nevertheless, more studies are warranted to provide more information on the possible mechanisms. In addition, a study in AIDS patients showed that the detrimental T_H_2-driven response caused by HIV infection can be transformed into a more beneficial T_H_1 response by the mixture of SIT and SITG, with a progressive decrease in plasma viral loads over time [221]. Considering that SIT or SITG do not have innate antiviral properties, the inhibition of viral duplication may be attributed to enhanced cellular immune function. It inspires us that SIT could be used to prevent certain cancers forerun by viral infections, such as cervical cancer and hepatocellular carcinoma. SIT effectively attenuated the expression of HPVE6 viral oncogenes in cervical cancer cell lines confirmed this from another side [35].

There are also notable studies that have argued the potential effects of SIT on other immune cells, such as mast cells [222] and monocytes [223]. Because these functions are less relevant to the anticancer activity of SIT, they are not discussed in this review.

### Modulation of COX-2

COX-2 is responsible for the production of prostanoid-like prostaglandin E_2_ (PGE_2_) and has long been known as a target for pain relief and treatment of inflammation [224]. Unlike its isoform COX-1, which mediates physiologic functions and is constitutively expressed in most tissues, COX-2 expression is negligible in normal cells but is induced to overexpress at inflammatory tumorigenic sites in most types of cancer [225,226]. Reciprocal positive feedback exists between COX-2 and inflammatory mediators, with one evoking the other [226]. In addition, COX-2/PGE_2_ released from cancer cells is able to induce an immunosuppressive state in the TME by recruiting immunocytes with inhibitive function or blocking the activity of cytotoxic T lymphocytes [227].

SIT significantly inhibited COX-2 activity without affecting COX-1 activity in colorectal carcinoma cell lines, indicating that SIT is a selective COX-2 inhibitor [41]. Similarly, by analyzing the dose-responsive transcriptional range of tumorigenic inflammatory genes after SIT treatment, researchers found that COX-2 expression and PGE_2_ concentrations were significantly decreased. The normalization of the COX-2/PGE_2_ tumorigenic inflammation axis by SIT inhibited the proliferation of colon cancer cells [37]. Moreover, SIT reduced the growth rate of premalignant esophageal cells, partly by downregulating COX-2 and decreasing prostaglandin production [132].

Various signals, such as ultraviolet radiation, carcinogens, and tumor promoter (TPA), stimulate *COX-2* transcription through the activation of mitogen-activated protein kinase kinase kinase (MEKK)/mitogen-activated protein kinase kinase (MAPKK)/MAPK-mediated, activator protein (AP)-1– mediated, p300-mediated, and inhibitory κB kinase/NF-κB–mediated signaling pathways [228]. Current studies have demonstrated that the inhibitory effect of SIT on COX-2 is mainly related to the NF-κB regulation of *COX-2* transcription and the MAPK pathway. The promoter region of *COX-2* contains binding sites for the redox-sensitive transcription factor NF-κB [224]. However, SIT can attenuate NF-κB activation in cancer cells as previously mentioned. Further mechanism studies found that, on the one hand, SIT inhibited the phosphorylation and degradation of IκB, the negative regulator of NF-κB that controls its release and nuclear translocation [229]. To the contrary, SIT may prevent the activation of NF-κB and the initiation of downstream transcription processes by regulating the redox state. This was further confirmed by a study in PMA-induced macrophages where SIT treatment simultaneously reduced ROS production and downregulated COX-2 expression [130]. The MAPK signaling pathway plays a complex role in the activation of COX-2. Proinflammatory cytokines such as IL-1β upregulate the expression of COX-2 by activating p38 MAPK and ERK1/2 [230]. In addition, both p38 and JNK induce phosphorylation of activating transcription factor 2, which allows its dimerization with the AP-1 transcription factor and thus association with COX-2 induction [231]. Therefore, the regulation of SIT on the MAPK signaling pathway, as previously mentioned, may also be one of the factors affecting COX-2 expression. Given that the COX-2–coordinated tumorigenic inflammatory TME has emerged as a new aspect of cancer therapy, SIT undoubtedly provides a promising additional option for selective COX-2 inhibitors.

### Modulation of other inflammatory mediators

In addition to regulating the balance of T_H_1 and T_H_2 cells with their corresponding cytokines and the COX-2/PGE_2_ signaling pathway, SIT has been shown to have effects on other inflammatory mediators. Unfortunately, these studies were not conducted in tumor cells but used other inflammatory diseases such as airway inflammation, arthritis, colitis, and obesity-related chronic inflammation as models. However, considering the extensive and universal role of inflammatory mediators and inflammatory signaling pathways in cancer and other diseases, it is necessary to mention the results of these studies. SIT has an ameliorating effect on airway inflammation in various diseases. In the rat model of asthma, SIT decreased the inflammatory factor concentrations of IgE, TNF-α, IL-6, TGFβ1, IL-5, IL-13, and IL-21 and increased the concentrations of IFN-γ and IL-10. In addition, SIT reduced the number of macrophages and neutrophils in the spleen and increased the number of NK cells and dendritic cells in peripheral blood, thereby inhibiting inflammation and improving asthma symptoms [232]. In another study of asthma, SIT improved airway inflammation and remodeling by binding to and upregulating glucocorticoid receptors, significantly suppressing type Ⅱ immune response (such as IL-25 and IL-33 secretion) and collagen deposition [233]. In the mouse model of lung chronic infection, SIT was proven to remarkably target the excessive neutrophil recruitment, thus favoring inflammation alleviation [234]. On the contrary, through modulating macrophage polarization, SIT contributed to the recovery of rheumatoid arthritis. In particular, SIT significantly repressed M1 polarization and inhibited the expression of iNOS, IL-1β, CD86, and MHCII whereas augmented M2 polarization and promoted the expression of IL-10, CD163, and CD206 [235]. Suppressing NF-κB to reduce the cytokines such as TNF-α, IL-1β, IL-2, IL-6, IL-16, and IL-17 and activating the HO-1/Nrf2 pathway is another mechanism by which SIT exhibited the antiarthritic effect [236]. It is widely accepted that obesity is associated with a state of chronic, low-grade systemic inflammation, whereas obesity also serves as a risk factor for several cancers, suggesting intrinsic links among them [237]. By inhibiting the increased expression of proinflammatory cytokines and the activation of NF-κB in the colon, SIT improved high-fat diet-induced colitis [238]. In addition, in patients with and without diabetes, the existence of rather specific negative correlations between the serum SIT concentration and the serum IL-6 and the TNF-α concentrations was observed. When *ABCG5/8* KO mice with significantly elevated plasma SIT concentrations were fed a high-fat diet, the plasma IL-6 and TNF-α concentrations, the expression of TNF-α in the liver and the expressions of IL-6 and TNF-α in the adipose tissue were lower [239]. These results suggest that SIT might suppress obesity-related chronic inflammation and might be applicable to the treatment of metabolic diseases and the prevention of cancer.

## Effects on multidrug resistance

Multidrug resistance (MDR) is one of the main obstacles in cancer chemotherapy. Cancer cells that are originally sensitive to a single chemotherapy later become resistant to multiple anticancer agents, leading to tumor recurrence or metastasis. Studies have indicated that there are 3 major mechanisms of MDR: first, the uptake of water-soluble drugs that require transporters to enter cancer cells is reduced; second, cancer cells undergo various changes that affect the killing capacity of cytotoxic drugs, such as alterations in cell cycle, enhanced DNA damage repair, reduced apoptosis, and changed metabolism of drugs; third, the energy-dependent efflux of hydrophobic drugs is increased [240]. Among these mechanisms, the most commonly encountered one is the increased efflux of anticancer drugs mediated by a family of energy-dependent transporters, known as ABC transporters.

The most extensively studied ABC transporters involve p-glycoprotein (P-gp, also known as ABCB1 or MDR1), multidrug resistance–associated protein (MRP) 1 (also known as ABCC1), and breast cancer resistance protein (BCRP, also known as ABCG2 or MXR) [240]. P-gp stands out among ABC transporters by conferring the strongest resistance to the broadest range of compounds. It delivers the central drugs of most chemotherapeutic regimens, including but not limited to vinca alkaloids, anthracyclines, epipodophyllotoxins, and taxanes [241]. In a recent observation, both tumors and cell lines derived from a ceritinib-resistant patient exhibited high concentrations of P-gp [242]. In addition, in vitro and in vivo resistance of P388/VCR cells to vincristine was reversible with verapamil, the first-generation P-gp inhibitor, which immediately suggested the possible therapeutic use of inhibitors to improve the efficacy of chemotherapy substrates of P-gp [243]. MRP1 is expressed in a wide range of clinical tumors and cancer cell lines and confers resistance to several hydrophobic compounds that are also P-gp substrates [244]. MRP1 homologs associated with MDR include MRP4, MRP6, MRP7, and MRP8. Cells transfected with MRP6 are resistant to etoposide, doxorubicin and daunorubicin, whereas MRP7 is a resistance factor for taxnanes [245,246]. BCRP is overexpressed in several drug-resistant cell lines and clearly has the potential to promote drug resistance. With wide substrate specificity, BCRP can transport methotrexate and some of the most recently developed anticancer drugs, such as tyrosine kinase inhibitors [247]. Cancer stem cells expressing high concentrations of BCRP defy treatment, serving as an unrestricted reservoir for tumor recurrence [248].

To date, 3 generations of inhibitors/modulators capable of reversing MDR have been developed. However, low potency and substrate specificity, high toxicity, and potential drug–drug interactions limit the application and clinical benefit of these inhibitors [249]. Compared with artificial compounds, natural compounds or plant extracts can act as potent inhibitors of MDR transporters with less cytotoxicity and better compatibility [250]. A breast cancer study showed that SIT exhibited strong cytotoxicity in both MCF7 cells and multidrug-resistant NCI/ADR-RES cells. Despite the higher cytotoxicity in the former that had basic expression of P-gp, SIT inhibited P-gp activity in both cell types, indicating that SIT was able to act as an effective P-gp inhibitor to target tumor cells with high MDR potential [251]. Another study on colorectal cancer reported evidence that SIT could activate p53 by disrupting p53-MDM2 (an E3 ubiquitin ligase inducing p53 ubiquitination and degradation) interaction, leading to increased translocation of p53 to the nucleus and silencing of the NF-κB pathway, which is essential for BCRP expression. This effect unsurprisingly blocked BCRP expression and subsequently recovered the sensitivity of oxaliplatin-resistant colorectal cancer to oxaliplatin, laying the foundation for the combination of SIT and oxaliplatin [252]. Loss of p53 function has been reported to activate the *MDR1* promoter [253]. It is reasonable to believe that SIT-mediated P-gp downregulation may be related to p53 activation as well. In addition, the localization and activity of BCRP are associated with cellular cholesterol content and lipid raft structure. Cholesterol depletion has been shown to reduce BCRP activity by 40% [254]. Therefore, altering cholesterol content of membrane lipid rafts may also be a mechanism by which SIT weakens BCRP-mediated MDR. On the contrary, it was confirmed by a competitive transport assay that SIT was not the substrate for P-gp, MRP1, or BCRP because the addition of SIT did not interfere with the efflux of their respective substrates [255]. Hence, it can be speculated that SIT is more likely to reverse MDR by reducing the content of MDR transporters rather than competing with anticancer drugs for MDR transporter-mediated efflux. Finally, SIT has a significant synergistic effect with gemcitabine, but it is uncertain whether this synergistic effect can effectively reverse drug resistance [53].

## Potential Connections Between SIT’s Anticancer Mechanisms

**FIGURE 7:**
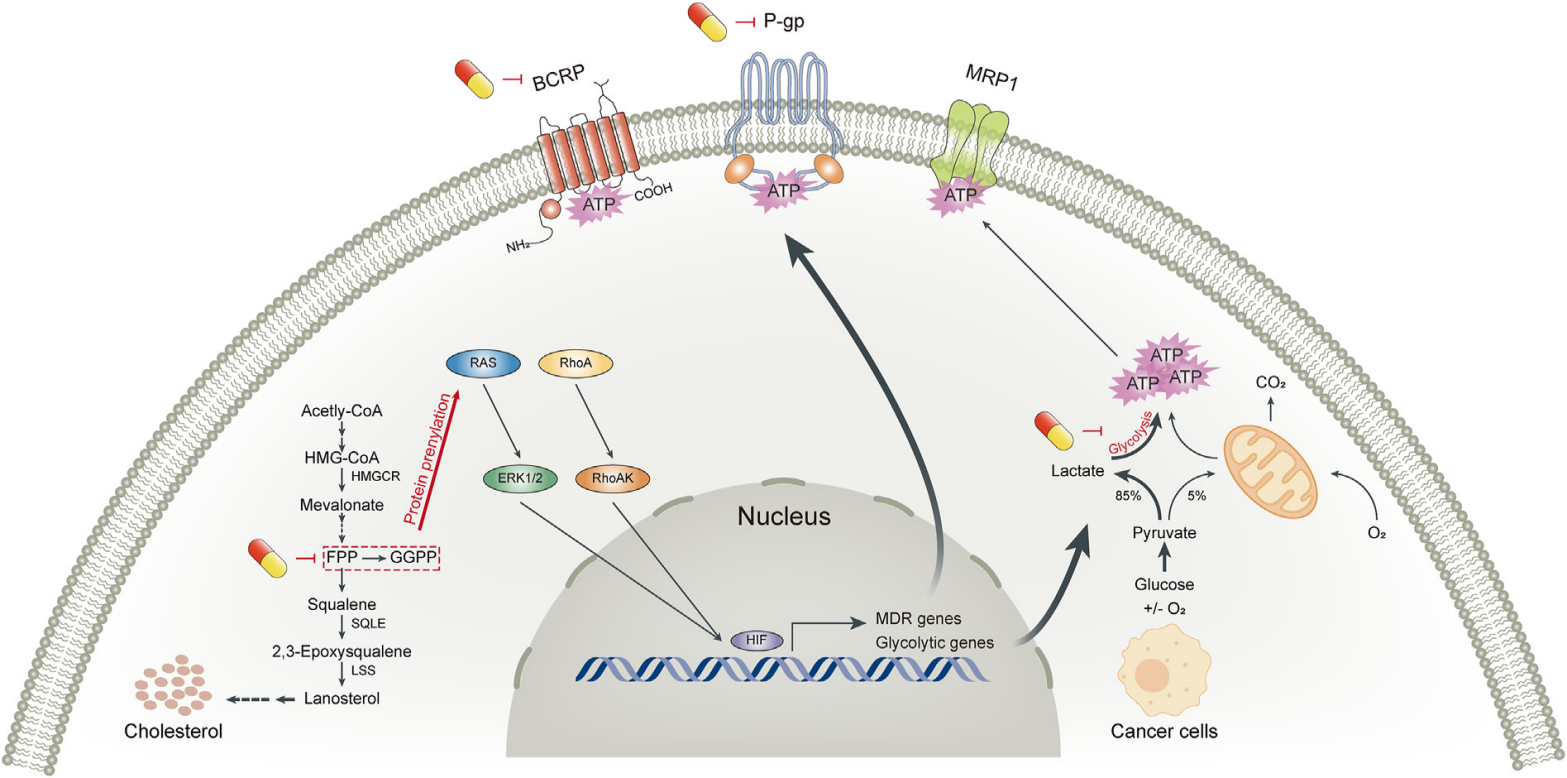
Active isoprenoid biosynthesis–induced MDR phenotype. Cancer cells, particularly drug-resistant cells, exhibit a high flux through the mevalonate pathway. The mevalonate pathway is the main pathway for cholesterol biosynthesis, and isoprenoid moieties, such as FPP and GGPP, are byproducts of this pathway. Farnesylation and geranylgeranylation determine the activation of Ras and RhoA proteins that engage their downstream transducers, ERK1/2 and RhoA kinase, respectively, to phosphorylate and activate transcription factor HIF. HIF-1α upregulates MDR1 and glycolytic genes, increasing the amount of P-gp and the amount of intracellular ATP produced by glycolysis. Consequently, the activity of several drug efflux transporters—P-gp, BCRP, and MRPs—is increased. This complex mechanism network is druggable by β-sitosterol (SIT), and these MDR transporters themselves can be directly inhibited by SIT as well. BCRP, breast cancer resistance protein; ERK, extracellular regulatory kinase; FPP, farnesyl pyrophosphate; GGPP, geranylgeranyl pyrophosphate; HIF, hypoxia-induced factor; MDR, multidrug resistance; MRP, multidrug resistance–associated protein; P-gp, P-glycoprotein.

As anticipated, SIT’s antitumor mechanisms can be summarized into several sections, corresponding to different critical aspects of tumor biological behavior. However, during the actual process of tumorigenesis and tumor progression, these different signaling pathways do not act independently but are interweaved and influence each other, jointly facilitating the malignant phenotype of tumor. Therefore, it is necessary to elucidate some of the connections between the different sections—although there is currently no evidence that SIT affects these connections—to pave the way for further exploration. Owing to the myriad of connections between different signal pathways, it is impossible to list all of them that may be related to SIT. In this review, we propose a paradigm to link cholesterol metabolism to other mechanisms because SIT is best known for its cholesterol-lowering effect (Figure 7).

The mevalonate pathway and its derivative isoprenoids are potentially related to MDR. An increasing number of evidence demonstrates that the mevalonate pathway is more active in cancer cells and promotes MDR by altering the intracellular concentration of isoprenoids such as FPP and geranylgeranyl pyrophosphate [256]. Indeed, zoledronate and alendronate, 2 inhibitors of isoprenoid synthesis, significantly reverse the MDR phenotype in glioblastoma multiforme and breast cancer [257,258]. FPP and geranylgeranyl pyrophosphate are used as substrates for protein prenylation. Multiple oncogenes, such as Ras, Rho/Rac/Cdc42, and Rab families are the isoprenylated proteins most involved in the process of prenylation [256]. As a downstream effector of Ras, ERK1/2 phosphorylates and stabilizes HIF in various cancers, including breast and lung cancers and chronic lymphocytic leukemia [[258], [259], [260]]. HIF-1α upregulates P-gp at transcriptional concentration [261], wheras HIF-2 activates *BCRP* transcription [262]. Rho family members and interactors are overexpressed in drug-resistant cancer cells where they contribute to the MDR phenotype by upregulating P-gp [263]. RhoA/RhoA kinase is also a parallel axis contributing to the phosphorylation and activation of HIF-1α apart from Ras [256]. Hence, a reduced activity of Ras/RhoA achieved with zoledronate and a targeted inhibition of ERK1/2 or RhoA kinase reverse the MDR phenotype. However, glycolysis also plays a role in this complicated mechanism network. In parallel to upregulating the MDR transporters, an active Ras/ERK1/2/HIF-1α axis increases the transcription and activity of glycolytic enzymes, thereby enhancing the ability to produce ATP via both aerobic and anaerobic glycolytic [256]. The resulting higher amounts of ATP provides enough fuel for ABC transporters, supporting the ATP-driven efflux of different chemotherapeutic drugs.

Several main aspects in this mechanism network, including the mevalonate pathway, ERK signaling pathway, aerobic glycolysis, and MDR transporters, have been verified to be targets of SIT in tumor cells. Given that the mechanism by which SIT reverses MDR is still not fully elucidated, the abovementioned connections may be an unproven mode of action. It is reasonable to assume that SIT reduces the production of isoprenoids and inhibits the Ras/ERK1/2/HIF or RhoA/RhoA kinase/HIF signaling pathways by inhibiting the mevalonate pathway, thereby downregulating the expression of MDR transporters such as P-gp and BCRP. Its hypoglycemic effect may play a synergistic role in this process. In addition, the mevalonate pathway and its byproducts have been reported to play an integral role in several other signaling pathways regulating cell proliferation and apoptosis, such as MAPK [264]. Therefore, these potential connections along with the mechanism network described earlier could be used as future research directions.

## Limitations and Improvements of SIT

Although a large number of in vitro and in vivo studies have confirmed the anticancer role of SIT on multiple tumors through a variety of mechanisms, there remain some obstacles and challenges that need to be addressed before SIT can be widely applied as an effective chemopreventive and chemotherapeutic agent. Principally, SIT has a low capacity for intestinal absorption, which, together with its high rate of biliary excretion, leads to a low concentration of bioavailability [265]. Hence, its biological activity is most likely to be manifested locally in the lumen of the large bowel rather than systemically. Second, because SIT is poorly soluble in water, problems exist regarding the development of administration methods and carriers [207]. Initially, SIT is administered in the form of powder and must be used in relatively large quantities for a significant effect. For example, high dosages (up to 25 to 50 g/d) of SIT are required to achieve a satisfactory serum cholesterol-lowering effect [266]. The poor aqueous solubility and bioavailability are closely intertwined, coupled with low targeting efficacy, limiting the therapeutic efficacy and clinical application of SIT.

In recent years, to improve its poor aqueous solubility and systemic bioavailability and low targeting efficacy, SIT has been formulated as nanoparticles. These SIT-based nanoformulations have been tried against cancers and have shown more promising efficacy. Karim et al. [267] fabricated folic acid–functionalized SIT-loaded alginate/chitosan nanoparticles (β-SIT-Alg/Ch-NPs-FA) with good stability and high drug encapsulation efficiency. Compared with SIT-suspension, β-SIT-Alg/Ch-NPs-FA exhibited higher solubility and intestinal permeability. Cell viability studies revealed that β-SIT-Alg/Ch-NPs-FA was more cytotoxic than SIT-suspension in breast cancer cell lines, probably owing to the nanoparticles protecting SIT from degradation and enhancing the tumor-targeting effect through FA receptors on the tumor cell surface. Andima et al. [268] prepared SIT-loaded poly (lactide-co-glycolic acid) (PLGA) nanoparticles (β-Sit-PLGA) using emulsion-solvent evaporation technique. β-Sit-PLGA was easily absorbed and able to release SIT in a sustainable manner. Compared with the conventional carrier vehicle, encapsulation of SIT into PLGA nanoparticles enhanced its antiproliferative activity against MCF-7 and MDA-MB-231 cells in a concentration-dependent manner.

On the contrary, some studies have used SIT as an excipient to construct nanoparticles or micelles loaded with other chemotherapeutic agents, indirectly using and promoting the antitumor effects of SIT. In these studies, high hydrophobic SIT forms amphiphilic conjugates with hydrophilic compounds, which greatly improves the stability of nanoparticles or micelles. The biodegradability of SIT also guarantees the safety of the drug delivery system. For instance, SIT-assisted silver nanoparticles (NPs) synthesized with SIT as a reducing and stabilizing agent showed a promising cytotoxic potential. SIT silver NPs caused apoptosis in HepG2 cells through the intrinsic apoptosis pathway by virtue of their intracellular ROS-inducing potential [121]. Cheng et al. [269] used the efficient self-assembly ability of SIT to construct SIT-NPs to deliver the photosensitizer chloride e6 (Ce6) for improved photodynamic therapy. SIT-Ce6 NPs possessed good biodegradability and biocompatibility with no noticeable side effects. In parallel, SIT-Ce6 NPs notably improved in vitro phototoxicity of Ce6 and the inherent anticancer activity of released SIT exerted a synergistic effect with photodynamic therapy. In another study, redox-sensitive (bioreducible) micelles (bHSC) were synthesized using heparin, SIT and cysteamine and encapsulated with doxorubicin (DOX) [270]; bHSC had strong stability, drug-loading capacity and blood compatibility and low toxicity. However, bHSC responded to high reduced GSH regions inside cancer cells to specifically release DOX. Its antimetastatic effects also made it a good candidate drug delivery system for the treatment of metastatic cancer. Similarly, stable pHPMA-DOX-SITO copolymer micelles were successfully synthesized by conjugating DOX and SIT to the *N*-(2-hydroxypropyl) methylacrylamide (HPMA) polymers and subsequently crosslinking side chains of the copolymer through hydrazone linkages [271]. pHPMA-DOX-SITO copolymer micelles were pH-sensitive and could release DOX in acidic environments such as lysosomal or endosomal compartments of tumor cells, with good antitumor activity and low toxicity.

In general, besides the poor aqueous solubility and bioavailability, SIT has no other outstanding application limitations. Meanwhile, SIT, as a compound of natural origin and rich in the diet, has been confirmed not to be genotoxic and mutagenic [50]. Therefore, SIT is safe for clinical use, but there are several known minor side effects. One of the side effects is that SIT interferes with the absorption of carotenoids. Intake of SIT results in a decrease in carotenoid concentrations in the blood, but this can be compensated for by diets with a high intake of carotenoids or small quantities of β-carotene [272]. In addition, excessive intake of SIT may lead to sitosterolemia, characterized by increased concentrations of phytosterols in the blood. Nevertheless, this is a rare genetic metabolic disorder caused by loss-of-function mutations in ABCG5 or ABCG8, both of which play important roles in the selective excretion of phytosterols [273]. In the normal population, there is little need to worry about this side effect when using SIT as an antitumor chemopreventive and chemotherapeutic agent. Regarding drug interactions, there are no reports indicating severe drug incompatibility for SIT. Moreover, current studies have found that SIT has synergistic effects with several drugs related to different diseases [[274], [275], [276]]. The synergistic effects of SIT with other drugs are summarized in Supplemental Table 2.

## Conclusion and Perspectives

As a phytochemical from natural sources, SIT has been demonstrated to possess outstanding potential as a cancer chemopreventive and chemotherapeutic agent in animal and cell culture studies. SIT can affect more than a dozen tumors in multiple systems, such as the respiratory (lung cancer), digestive (liver, colon, stomach, and pancreatic cancers), hematological (leukemia and multiple myeloma), nervous (fibrosarcoma), and genitourinary (prostate, cervical, and breast cancers) systems, so the potential breadth of its anticancer effects is considerable. SIT inhibits multitude of pathways involved in carcinogenesis and cancer development and has not shown any evidence of toxicity. In light of the studies reviewed in this study, there are at least 7 major aspects of SIT’s antitumor mechanisms of action, which are intertwined and implicated in almost all common cancer types. Therefore, sufficiently focused, powered, and well-designed randomized controlled trials to evaluate SIT as a clinical cancer prevention or treatment agent are valuable and urgently needed. Although SIT holds such great promise, the current pace of research on SIT has slowed down significantly, thus making SIT an orphan nutraceutical [11]. Part of the reason is that the poor bioavailability and low targeting effect limit its extensive application. The use of drug delivery systems can improve this deficiency to some extent. Encapsulation of SIT using NPs can help to concentrate SIT at the disease site through the targeting ability and the enhanced permeability and retention effect, thereby improving its anticancer efficacy. Overall, the continued investigation into SIT holds great promise for optimizing SIT for future use as a dietary cancer chemopreventive and chemotherapeutic agent. Our review of convincing results from in vitro and in vivo studies warrants the design of clinical studies to better understand the relationship between SIT and cancer. On this ground, we appeal that SIT deserves more attention in an academic setting. Additional experiments should be designed to discover the anticancer mechanisms of SIT more profoundly, and further research could be conducted on how to overcome its relatively lower efficacy with modern drug delivery systems.

## Acknowledgments

The authors’ responsibilities were as follows—LH, HYW: designed the review outline; HYW, ZW, ZHZ, JCL: sourced literature; HYW: wrote the paper and prepared the figures; HYW, ZW, ZHZ, JCL, LH: reviewed and revised the manuscript; LH: had primary responsibility for final content; and all authors: read and approved the final manuscript.

## Appendix A Supplementary data

Multimedia component1 Multimedia component1
