# Supplementary material for: β-Sitosterol as a Promising Anticancer Agent for Chemoprevention and Chemotherapy: Mechanisms of Action and Future Prospects
Source: Adv Nutr. 2023 May 27;14(5):1085–110. doi: 10.1016/j.advnut.2023.05.013 (PMC10509430; doi:10.1016/j.advnut.2023.05.013)
Supplement: Multimedia component1 [file mmc1.docx]

**Beta-sitosterol as a promising anticancer agent for chemoprevention and chemotherapy: mechanisms of action and future prospects**

**Haoyu Wang**

**Supplementary Table 1.** ***In vitro* studies highlighting SIT-induced cell cycle arrest.**

| **Cancer type** | **Cancer cell line(s)** | **Arrested phase** | **Mechanisms** |
| --- | --- | --- | --- |
| Breast carcinoma(44) | MDA-MB-231 | G0/G1 | cyclin D-CDK4 (↓),  p21 (↑), p27 (↑) |
| NSCLC(49) | A549 | G0/G1 | cyclin D-CDK2 (↓),  p21 (↑), p53 (↑) |
| Breast Cancer(85) | MDA-MB-231 | G2/M | cholesterol (↓),  CDK1 (p34/Cdc2) (↓) |
| Cancers of the oral cavity and  pharynx(84) | SCC-83-01-82 (premalignant),  83-01-82CA (malignant) | G0/G1 (SCC-83-01-82)  G2/M (83-01-82CA  ) | cyclin B-CDK1 (↓),  cyclin D (↓),  p21 (↑) |
| Prostate cancer(87) | PC-3 | G2/M | Unknown |
| Lung cancer(50) | A549 | G2/M | Unknown |
| Colorectal cancer(41) | HT-29 | G0/G1 | Unknown |
| Pancreatic cancer(53) | MIAPaCa-2, BXPC-3 | G0/G1 | Unknown |
| NSCLC(76) | A549 | G0/G1 | Unknown |
| Leukemia(99) | U937, HL60 | G2/M | α-tubulin (↑),  a promotion of tubulin polymerization,  p21 (↑), CDK2 (↑) |
| Cervical cancer(101) | SiHa | S | α-tubulin (↓),  microtubule-associated protein 2 (↓), an inhibition of microtubular polymerization |

CDK, cyclin-dependent kinase.

**Supplementary Table 2.** **The synergistic effects of SIT with other drugs**

| SIT | Disease(s) | Synergistic drug(s) | Synergistic effect(s) |
| --- | --- | --- | --- |
|  | Melanoma | Vemurafenib(51) | Reducing mitochondrial respiratory capacity. |
|  | Pancreatic cancer | Gemcitabine(53) | Modulating apoptosis and inhibiting EMT. |
|  | Breast cancer | TRAIL(68) | Activating caspases. |
|  | Breast cancer | Tamoxifen(177) | Raising cellular CER content. |
|  | Anxiety and stress-related conditions | Fluoxetine(274) | Reducing the effects of restraint stress, contextual fear memory, and c-Fos activation in the prefrontal cortex and dentate gyrus. |
|  | Cardiovascular disease | Atorvastatin, ezetimibe(275) | Improving lipid profile, without additional effects on intestinal sterol absorption or synthesis. |
|  | Vitamin D deficiency, autoimmune diseases | Vitamin D(276) | Enhancing the action of vitamin D on the immune function of macrophages. |

EMT, epithelial-mesenchymal transition; CER, ceremide.
